# Spatial quorum sensing modelling using coloured hybrid Petri nets and simulative model checking

**DOI:** 10.1186/s12859-019-2690-z

**Published:** 2019-04-18

**Authors:** David Gilbert, Monika Heiner, Leila Ghanbar, Jacek Chodak

**Affiliations:** 1 Department of Computer Science, Brunel University London, Uxbridge, UB8 3PH UK; 2 Computer Science Department, Brandenburg University of Technology Cottbus-Senftenberg, Cottbus, D-03046 Germany

**Keywords:** Quorum sensing, Biofilm formation, Coloured hybrid Petri nets, Coloured stochastic Petri nets, Diffusion in 3D space, Simulative model checking

## Abstract

**Background:**

Quorum sensing drives biofilm formation in bacteria in order to ensure that biofilm formation only occurs when colonies are of a sufficient size and density. This spatial behaviour is achieved by the broadcast communication of an autoinducer in a diffusion scenario. This is of interest, for example, when considering the role of gut microbiota in gut health. This behaviour occurs within the context of the four phases of bacterial growth, specifically in the exponential stage (phase 2) for autoinducer production and the stationary stage (phase 3) for biofilm formation.

**Results:**

We have used coloured hybrid Petri nets to step-wise develop a flexible computational model for *E.coli* biofilm formation driven by Autoinducer 2 (AI-2) which is easy to configure for different notions of space. The model describes the essential components of gene transcription, signal transduction, extra and intra cellular transport, as well as the two-phase nature of the system. We build on a previously published non-spatial stochastic Petri net model of AI-2 production, keeping the assumptions of a limited nutritional environment, and our spatial hybrid Petri net model of biofilm formation, first presented at the NETTAB 2017 workshop. First we consider the two models separately without space, and then combined, and finally we add space. We describe in detail our step-wise model development and validation.

Our simulation results support the expected behaviour that biofilm formation is increased in areas of higher bacterial colony size and density. Our analysis techniques include behaviour checking based on linear time temporal logic.

**Conclusions:**

The advantages of our modelling and analysis approach are the description of quorum sensing and associated biofilm formation over two phases of bacterial growth, taking into account bacterial spatial distribution using a flexible and easy to maintain computational model. All computational results are reproducible.

**Electronic supplementary material:**

The online version of this article (10.1186/s12859-019-2690-z) contains supplementary material, which is available to authorized users.

## Background

*Human gut microbiota.* The human body hosts a large number of prokaryotic and unicellular eukaryotic cells, which are found in most tissues. This huge population can be up to 100 trillion in the human body, almost the same number as human cells, with the total mass of around 0.2 kg [1].

Human gut microbiota play an important role in human health and are necessary for a human to survive. For instance, human gut microbiota help in vitamin synthesis, dairy digestion, gut development, nutrient processing, resistance to pathogens, brain development and function, immune cell development and immune responses [2]. It is notable that some of these bacteria in human intestine can prevent certain diseases such as diabetes [3] or liver diseases [2]. In addition, gut microbiota have a direct effect on the diseases related to intestines such as Irritable Bowel Syndrome (IBS), Inflammatory Bowel Disease (IBD) [2], metabolic diseases and obesity [4].

The composition of gut microbiota is unique for each individual host. However, family members can have more similar gut microbiota compared to unrelated people. The composition of gut microbiota is influenced by diet, drugs, lifestyle, stress and genetics [2, 4].

*Quorum sensing.* Bacteria need to act in a concerted manner in order to achieve large scale effects in, for example, a multicellular host such as human. This involves communication, and the detection of the presence of other bacteria of the same species or strain whose activities can be coordinated and aggregated. This process of stimulus and response correlated to population density is called quorum sensing which can happen among microbes, particularly among the bacteria of the same strain, with other strains and with human cells [5]. This communication mechanism between cells is based on chemical broadcasting, i.e. one-way communication whereby molecules acting as chemical signals are transmitted via diffusion without requirement for an acknowledgement of receipt. The concentration of the molecules involved stands as a proxy for population density. The receiving cells detect these molecules via their membrane receptors which employ a threshold mechanism over the concentration of the molecules. This results in the activation of pathways which induce changes in gene expression.

Bacteria use quorum sensing to initiate some changes in their behaviour or phenotype, such as biofilm formation, pathogenicity and virulence factor production, motility and toxin production. These changes play an important role in the relationship between the host and the microorganism [6].

This process is achieved by the production, release and detection of a signalling molecule called autoinducer (AI) [7]. According to Elias and Banin, distance has an impact on the ability of the cells to intercommunicate. Their study shows that the distance between the cells and hence the compactness of the society of the bacteria is a more important factor than the size of the population itself [8]. When the population density is low, the total amount of AI produced is too low for the quorum sensing process to be enabled. As soon as AI is produced and exported, it diffuses into the environment. Thus it can not be detected by the individual cell to activate biofilm formation. An increase in the population increases the amount of AI observable in the intercellular environment, facilitating the ability of the cell to detect and import it. AI accumulates in the cell and can activate transcriptional signals by binding to a regulator protein which is a gene transcriptional suppressor. This is when quorum sensing is switched on [6].

**Fig. 1:**
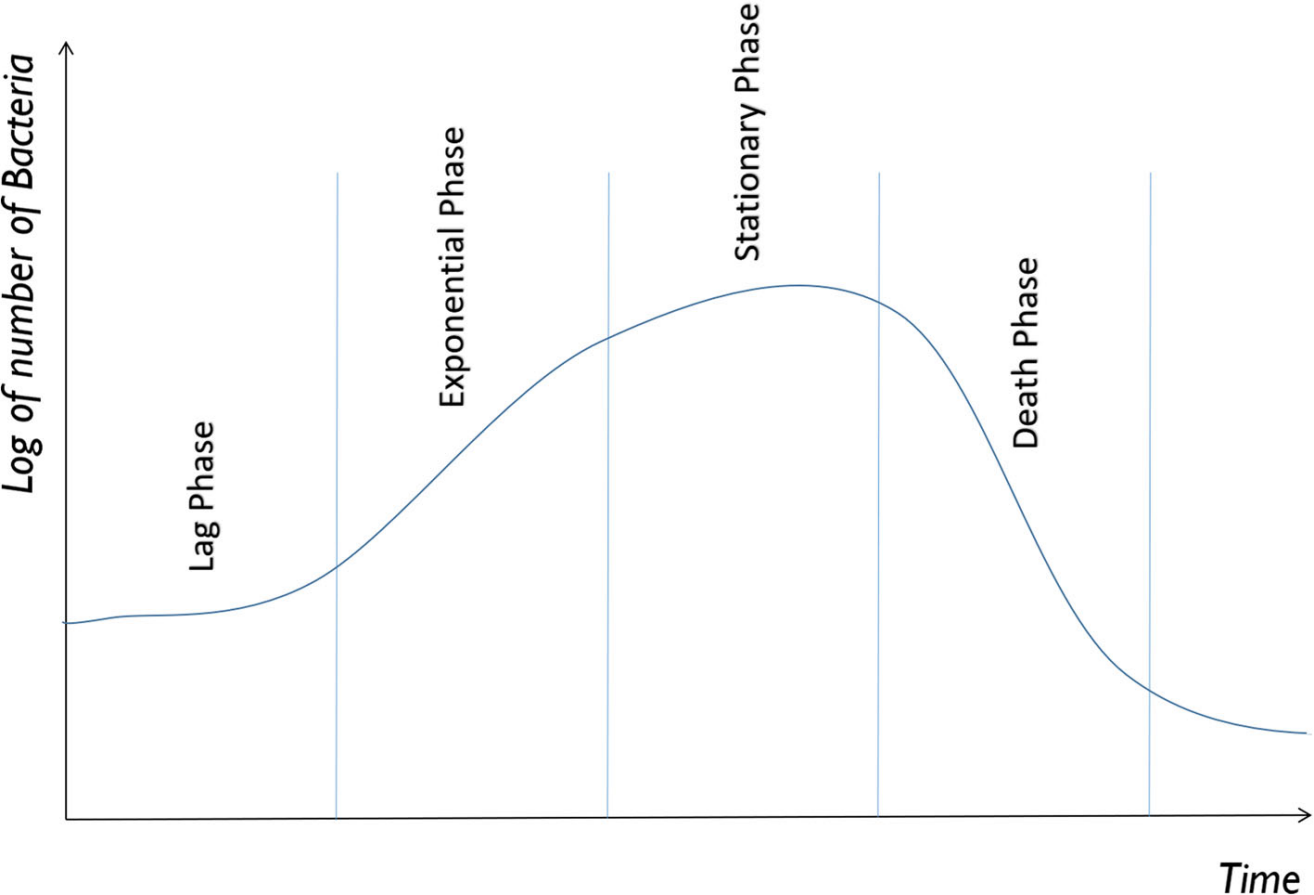
Bacteria population growth phases. Our model relates to the Exponential and Stationary Phases

Bacterial population growth occurs in four different phases, see Fig. 1. In the first stage, Lag Phase, the bacteria adapt to the environment, and growth is not fast. In the second stage, Exponential Phase, the bacteria start to grow fast such that their number doubles logarithmically. In the third stage, Stationary Phase, the growth rate and death rate of bacteria equalise due to limited availability of growth factors and the accumulation of waste products. The final Death Phase takes place when waste products dominate the environment and the growth rate dramatically decreases while death rate is high. Autoinducer production occurs in the Exponential Phase (Phase 2) and the response of the bacteria to it occurs in the Stationary Phase (Phase 3) [9].

Looking at the phases of bacterial growth in detail, AI is produced and exported to the intracellular environment during the mid-Exponential Phase and early Stationary Phase. In the case of a sufficiently large population of bacteria, the level of AI exceeds its threshold, which happens during the late Phase 2 and the start of the Phase 3, when the bacteria start to import this chemical to their cytoplasm [10]. At this point (Phase 3), AI production is slow while the import is so rapid that no AI in the environment is detectable [11].

In Gram-negative bacteria, there are three classes of AI according to the way they are produced: AI-1, AI-2, AI-3 [12]. The main focus of this study is on Autoinducer-2 (AI-2) which is the only AI molecule that is produced and detected by both Gram-negative and Gram-positive bacteria [10]. AI-2 is synthesized by LuxS or its homologues from methionine and is actively transported to the extracellular environment throughout cell growth [12, 13].

When non-pathogenic *E.coli* produce AI-2 in the intestine, an inflammatory response is initiated which is quickly arrested. Not only can AI-2 act as an inter-kingdom signal between bacteria and their hosts [12], but it is also non-specific between bacterial species. Thus when the AI-2 system is activated, the signal molecule is quickly removed from the environment by absorbtion by the receiving cells in order to avoid cross-talk with other strains of bacteria. This mechanism prevents these strains from using AI-2 to change their behaviour — for example, interacting inappropriately with the host. In *E.coli*, AI-2 affects virulence factors, motility, pathogenicity and biofilm formation [12]; in this paper we concentrate on the latter.

*Biofilm formation.* Quorum sensing can cause changes in behaviour and morphology of a bacterium. One of the consequences is the formation of biofilm. Biofilm formation is a structural process where a polymer layer of carbohydrates or small amounts of protein and DNA, are produced and exported to the micro-environment [14]. This process results in phenotype changes while enabling the bacteria to adhere to each other and the surface.

The bacteria in this community are structurally organised by the way in which they communicate with each other and respond to one another’s signals. The formation of biofilm can have several beneficial effects for bacterial colonies: it (i) can act as a physical protection against antibiotics, thus facilitating bacterial antibiotic resistance, (ii) helps them in colonization of the host by facilitating adherence of the bacteria to host tissues, (iii) is helpful in collecting nutrients from the environment [15], and (iv) facilitates DNA exchange between bacteria in the biofilm which results in changes in DNA recombination or DNA repair [14].

Biofilm production leading to inter-cell matrix formation does not form in sparse or scattered colonies because the distances between the bacteria are too high to permit high enough autoinducer concentration to pass the threshold required to trigger the relevant internal pathways [16].

The study of biofilm is an important field for microbiologists and immunologists since it can be a threat for human health. Biofilms are involved in 65% of hospital infections, causing serious problems due to their high resistance to antibiotics [15].

In a microbiology lab, one of the constraining resources is time. In order to obtain results a researcher should wait at least for 24 h for a sufficient population of bacteria to have developed. By simulating biological systems in-silico it is possible to predict and analyse the behaviour of biological systems faster. This method, aside from being less time and money consuming, gives us a clear detailed result to study and to guide lab experiments.

### Related work

The first description of quorum sensing was given in the early 1970s for *Vibrio fischeri* [17, 18] and many scientific descriptions have subsequently been published; see the review by Waters and Bassler [19] for an overview of this large area.

Many mathematical models of the quorum sensing mechanism have been proposed, and are reviewed in depth in [16, 20]. Models of the intracellular molecular mechanism at the single cell level have mostly been continuous, using ordinary differential equations (ODEs). Some models are stochastic, for example Weber and Buceta [21] give both a deterministic model to describe AI-2 production in *E.coli* which treats the cell as one entity, and a stochastic model which divides the cell into individual compartments, including a noise term on the luxR gene expression, which depends on the cell density and may influence phenotypic changes stochastically. Another set of models describe self-controlling mechanisms in the quorum sensing process, where the general purpose of AI systems is held to be (i) the homeostatic control of costly cooperative behaviours [22] and (ii) that bacteria have evolved mechanisms to repress certain components of quorum sensing if needed, see e.g. [23].

One of the most complete works in this field is the stochastic Petri net model of *E.coli* AI-2 production, constructed by Li and his colleagues [24]; it has quantified values for the reaction rate constants and the initial concentrations of species. This model describes the AI-2 production pathway as outlined in “Phase 2 model – AI-2 production” section, which forms one component of our overall model.

The modelling of biofilms has a long history over the last 30 years, and has been extensively reviewed in [25]. The representation of space is a critical component of such models, and initially models comprised partial differential equations (PDEs) modelling a biofilm as a flat layer; subsequently multidimensional models have been developed to describe non-uniformities.

The mechanism of biofilm production itself has been described in papers by Li et al. [26] and Novak et al. [27]. However the descriptions are textual only without any quantified values for concentrations or rate constants, and no formal models are given.

Janowski et al. [28] have modelled both the production of autoinducer signals as well as their diffusion and cellular import in a quorum sensing scenario using standard Petri nets. They achieve a notion of space by constructing a large model explicitly comprising several copies of the Petri net descriptions of individual cells, connected by the diffusion of the AI molecules outside the cell. One drawback of this modelling approach is that each copy of the cellular mechanism needs to be updated if the intracellular description is changed during model development, and also the extracellular connections are explicitly represented individually and likewise would require individual updating.

Other approaches to describing quorum sensing at the population scale include agent-based models which permit the allocation of a range of conditions to different individuals. For example, Müller et al. [29] have proposed a spatial single-cell model of quorum sensing using two layers: intra-cellular and inter-cellular, and a population of such single cells, where the quorum sensing signalling system is modelled by nonlinear ODEs and PDEs are used to give spatial structure. P systems exploit a hierarchical membrane approach for modelling and have been used by Pérez-Jiménez and Romero-Campero [30] to describe quorum sensing in terms of an agent-based model where an individual agent or cell is described by a P system exploiting Gillespie-style [31] stochastics.

### Our contributions

We developed a spatial model describing *E.coli* biofilm formation driven by the Autoinducer 2 (AI-2) covering Phase 2 and Phase 3 of bacterial growth and comprising different abstraction levels. The model is represented as coloured Petri net, which can be equally read as stochastic or hybrid Petri net. We investigate different model configurations comprising the single bacterium, dense colony and sparse colonies of different sparseness, by simulating them with the approximative delta-leaping stochastic simulation algorithm [32].

We describe in detail our step-wise model development and validation strategy. All results are reproducible. Our approach of encoding space is flexible, it can easily be configured for the 1D, 2D or 3D scenario, and conveniently adjusted to different notions of space, such as different boundary conditions or neighbourhood relations [33]. Our modelling strategy can be equally applied to other problems evolving in time and space.

*Outline.* This paper is organised as follows. In the next section we describe our methods, comprising standard Petri nets in the stochastic, continuous and hybrid paradigms, as well as their coloured counterparts, and simulative model checking of linear time temporal logic properties. Next, we present our model and its step-wise development and validation; first we consider the model components separately without space, and then combined, and finally we add space. We conclude our paper with a discussion and conclusions suggesting further work. We also provide Additional files with supplementary material illustrating in more details our methodology and results.

## Methods

### Petri nets

To simulate a model in silico, a powerful tool is needed, which is able to show the complex interactions within a cell [34] in the simplest way possible. At the same time, this tool should be easy to work with and provide the required data such as graphs or data tables to be analysed. We chose to use Petri nets as they support a graphical and mathematical environment in silico; they ease communication among professionals with a diverse background and permit to computationally simulate a biological system.

Standard Petri nets are inherently discrete and free of any notion of time, which means that they consider behaviour under any timing constraints, supported by a substantial body of Petri net theory [35]. They have been proven to be useful for a wide range of applications, among them biochemical networks, such as metabolic networks, gene regulatory networks, or a combination of them [36]. If required for clarity, we call them qualitative Petri nets (QPN).

Over the years, Petri nets have been extended in various ways, for example by assigning firing rates to transitions, which can either be read as stochastic or deterministic rates. Stochastic rates yield stochastic Petri nets (SPN), which can be seen as high-level descriptions of continuous-time Markov Chains [37], while deterministic rates yield continuous Petri nets (CPN), which in turn can be seen as a structural descriptions of ordinary differential equations (ODEs) [38, 39]. Combining stochastic and continuous rates within one model brings us to hybrid Petri nets (HPN), which contain SPN and CPN as proper subsets [40]. Firing rates are often state-dependent and follow specific kinetic laws. In the case study considered in this paper, all firing rates follow mass-action kinetics [41].

We use the Petri net tool Snoopy [42] which supports Petri nets in different paradigms – as required for the case study discussed in this paper, comprising QPN, SPN, CPN, and HPN, as well as their coloured counterparts, see next section. Importantly, it is possible to conveniently convert the individual net classes into each other according to the user’s need.

In the following we assume basic knowledge of Petri nets; see [43] for a gentle introduction in the context of Systems Biology, and [44] for formal definitions.

### Coloured Petri nets

Enriching Petri nets by a colouring concept yields a kind of high-level Petri nets. Colours are organised in *colour sets*, which can be seen as a synonym for (finite) discrete data types as known from traditional programming languages.

Coloured Petri nets allow for concise descriptions of similar network structures, which in turn permits, among others, to conveniently encode space, as we have first shown in [45] and later applied for various notions of space, for example to deal with planar cell polarity requiring the nesting of two spatial concepts [46], or to investigate phase variation in bacterial colonies, where we applied Cartesian and polar coordinates [47]. The tutorial-like paper [33] showcases the use of coloured continuous Petri nets to encode partial differential equations.

A special strengths of this approach of colouring space is its flexibility: models can be conveniently adjusted to different notions of space, such as different boundary conditions or neighbourhood relations [33]. As we will see, models can also be designed in such a way that they subsume as special cases the one-dimensional, two-dimensional or three-dimensional settings. Thus, the basic modelling strategy can be applied to a large variety of problems evolving in time and space. See [48] for a recent review paper illustrating the wide use of coloured Petri nets for multi-level, multiscale, and multi-dimensional modelling of biological systems.

Coloured Petri nets consist, as standard Petri nets, of places, transitions and arcs. Additionally, a coloured Petri net is characterised by a set of discrete data types, the colour sets, and related net inscriptions.

- Places get assigned a colour set and may contain a multiset of distinguishable tokens coloured with a colour of this colour set.
- Transitions get assigned a guard, which is a Boolean expression. The guard must be evaluated to true for the enabling of the transition.
- Arcs get assigned an expression; the result type of this expression is a multiset over the colour set of the connected place.

We consider diffusion in 3D space to recall the basic concepts of coloured Petri nets, as supported in our toolkit in different paradigms, i.e. as coloured Petri nets (no notion of time), coloured stochastic Petri nets (coloured SPN), coloured continuous Petri nets (coloured CPN), and coloured hybrid Petri nets (coloured HPN).

In order to encode spatial information we introduce the following.

- Constants D1, D2 and D3: integers which define the length of the X, Y and Z axes of the environment space.
- Constant D: an integer to be used to define uniform lengths of the non-zero axes. For example, in order to model a 3D cube, they are all set to the same value D. For a square 2D grid, D1 and D2 are set to D while D3 is set to 1, and for a 1D linear array, D1 is set to D while both D2 and D3 are set to 1.

Having these constants we can now define the following colour sets specifying a 3D grid, and a colour function specifying the neighbourhood relation over the grid positions, which are triples of the X, Y, Z coordinates.


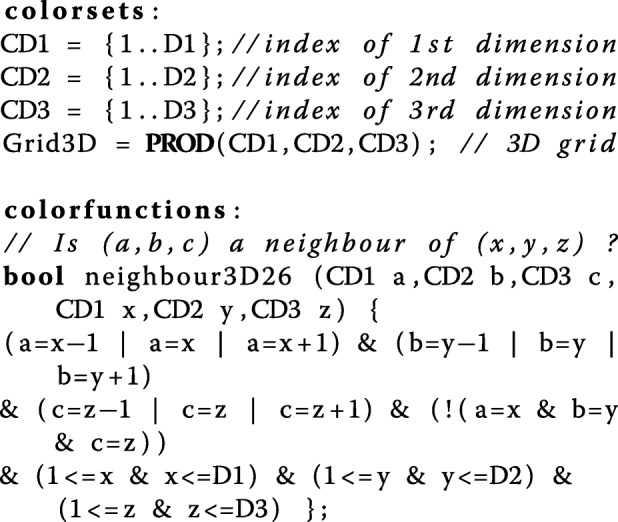


**Fig. 2:**
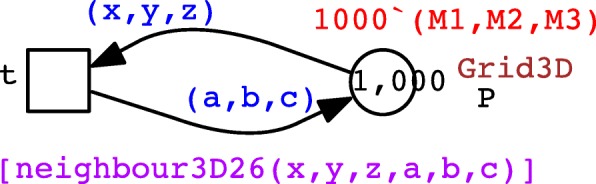
Diffusion as coloured Petri net. The initial marking sets 1000 tokens in the centre of the grid, specified by the constants *M*1, *M*2, and *M*3. The transition rate (not shown) follows the mass-action law with the kinetic parameter *k*.The size of the underlying unfolded Petri net is determined by the constants *D*1, *D*2, and *D*3; see Additional file 1: Figures S2–S4 for some examples. Additional file 1 also gives a complete self-contained textual specification of this coloured Petri net

The advantage of this approach relying on colour is to concisely define a potentially large model which has many repeated elements, one example of which is a spatial grid whose elements can be addressed by colour tuples specifying the grid positions, and movement in space translates into re-colouring of tokens. See Fig. 2 for a Petri net showing diffusion in 3D space which can be easily configured for 1D or 2D by merely changing the constants D1…D3. This coloured representation can be automatically unfolded generating the corresponding uncoloured Petri net in standard notation amenable to simulation and analysis techniques available for uncoloured Petri nets. See Additional file 1: Figures S2–S4 for some examples of unfolded nets.

We can further define a sub-space, called ‘region’ for brief, within the overall environment, using the following constants:

- Constants M1, M2 and M3: integers representing the central position of the X, Y, Z dimensions of the environment,
- Constant R: an integer controlling the radius of a region in each dimension (assuming a circular shape which maps to a rectangle in our representation). Note that when R=1, the side of the region is of length 3 and hence a 2D region comprises 3×3=9 grid positions and a 3D region comprises 27 positions. When R=2, the side is of length 5 giving 25 positions in the 2D case and 125 in the 3D case, and so on.
- Constants S1, S2 and S3: integers controlling the sparseness (density) of the bacterial colony in the X, Y and Z dimensions, respectively, where S _*i*_=1 results in no gaps in the region, S _*i*_=2 generates a region spread s.t. there is one empty grid position between each occupied position, S _*i*_=3 gives 2 empty grid positions, etc.

This allows us to introduce the following colour set and colour function:


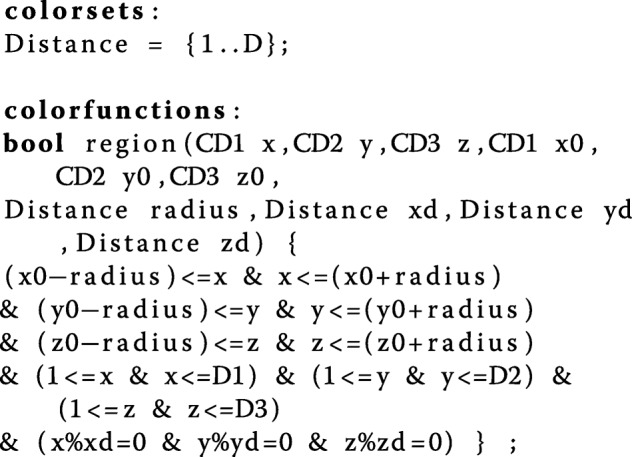


which defines a region around the centre (x0,y0,z0) with a max distance from the centre of ‘radius’, and a space of xd, yd, zd in-between in the X, Y, Z axes, respectively. In our current scenario, the main grid represents an environment within which a bacterial colony is located using the region function.

The entire diffusion mechanism with its associated colour definitions will be one of the three components of our final model, discussed when adding space – see “Adding space” section. A complete self-contained description is given in the Additional file 1.

### Temporal logics

Model checking can be used to determine whether the behaviour of a model conforms to some desired properties specified in temporal logic. We use here Probabilistic Linear-time Temporal Logic with numerical constraints (PLTLc) [49], based on Linear-time Temporal Logic (LTL) [50], extended with probabilities [51] and numerical constraints over real value variables [52]. Several features of PLTLc facilitate the expression of the behaviour of biochemical networks, including the ability to express properties relative to an absolute time value or range, the use of functions which compare the concentration of a protein to its peak value, and the derivative function enabling the description of transient, sustained or oscillatory behaviour.

The operators of PLTLc include the usual first order logic connectives: & (And), ∨ (Or), ¬ (Not), and → (Implication). In addition the language includes the following temporal operators over formulae: *F*(*ϕ*) (*ϕ* holds eventually (finally)), *G*(*ϕ*) (*ϕ* holds forever (globally)), *ϕ*_1_ *U* *ϕ*_2_ (*ϕ*_1_ holds until *ϕ*_2_ holds).

In this research we have used simulative model checking over time series traces of the species in the model which include the small molecules, metabolites, proteins, RNAs, genes and complexes. This can be done for single output of a continuous model, or for the several runs of a stochastic model – either separately or averaged. In our case, the behaviours that we check can also be generated by a hybrid model yielding both deterministic traces (for the continuous places) as well as stochastic traces (for the discrete places). We give the properties in PLTL format following the usage in the simulation-based MC2 model checker [49] which we have employed, with the results belonging to the set {0,1} rather than in being in the range [0..1], when applied to continuous or averaged traces.

MC2 has two built-in functions, the first of which, *max*, operates over all the values of a species to return the maximum of the species’ value in the simulation run, thus the peak of a species can be expressed using *max*(*Protein*). The other function *d* returns the derivative of the concentration of the species at each time point, thus increasing and decreasing species values can be expressed as *d*(*Protein*)>0 and *d*(*Protein*)<0, respectively.

Commonly, the property of interest refers to known values, often motivated by observations in the wet lab; e.g. *The concentration of metabolite A is always below a certain threshold, for example 100, and always decreasing.* This can be expressed by the temporal logic property:

*P*_≥1_[ *G*(*A*<100 & *d*(*A*)<0) ].

In the research reported in this paper we often are presented with a large number of species, for example all those in the particular network of interest in one bacterium, and would like to know which of these fulfil a particular property, then we can express the previous property by:

*P*_≥1_[ *G*(*$x* <100 & *d*(*$x*)<0) ], where $*x* is a meta variable ranging over all species in the model. This feature can also be applied to check all the species in the often very large models generated by uncolouring a coloured model by unfolding. As result we obtain the set of all variables fulfilling the given property, with the set being empty, when no variable fulfils the property.

## Results - models

In this section we show how our model has been developed using a step-wise approach. First we consider AI-2 production (Phase 2 model) and biofilm formation (Phase 3 model) separately as two models without space, then we combine them, and finally we add space to the combined model. Each step involves model validation.

### Phase 2 model – AI-2 production

**Fig. 3:**
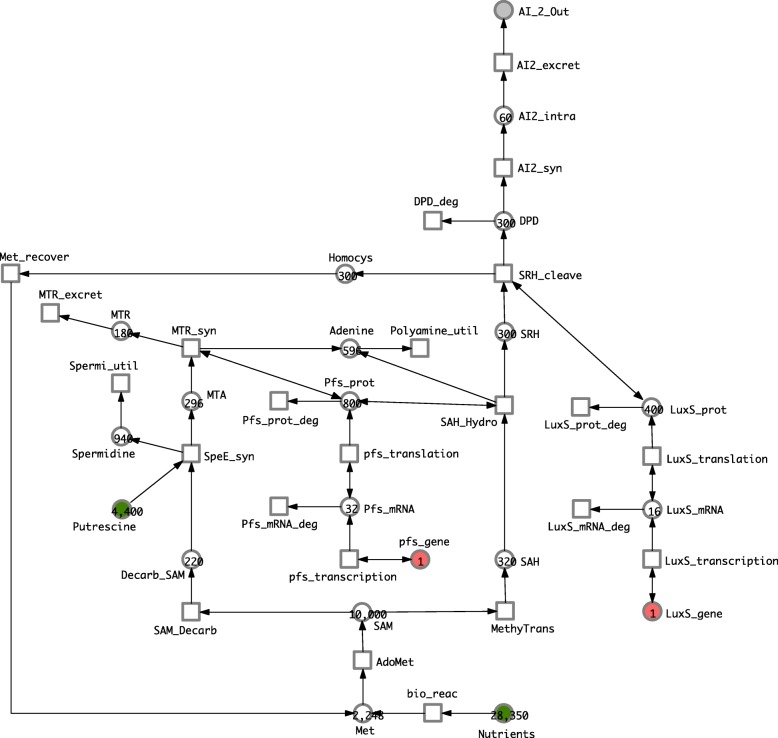
Phase 2 model. Petri net obtained by SBML import of the model presented in [24], mimicking the original layout, but rotated by 180 degrees. Note that we have removed the two transitions describing the phosphorylation of AI-2 and the transportation of AI-2 back into the cell since they occur in the Phase 3 biofilm formation part. All transition rates follow mass-action kinetics with specific rate constants (not shown); see Additional file 2 for details. Colour code: source places highlighted in green and genes in red. This model can be equally read as SPN or CPN

We have used the non-spatial stochastic Petri net model of AI-2 production given in Li [24] to describe the production of AI-2 in Phase 2, keeping the assumptions of a limited nutritional environment. The model does have elements of AI-2 activity in Phase 3, which we separate out into our description of biofilm formation (see “Phase 3 model – biofilm formation” section). The model is available in SBML format from the website of the PMC journal where the paper was published. By importing this SBML specification, we obtained the Petri net model shown in Fig. 3, which can be read as a stochastic Petri net or a continuous Petri net alike.

The detailed mechanism of the AI-2 production pathway is as follows: AI-2 is derived from S-adenosylmethionine (SAM) which is a methyl donor for the methyl transferase enzyme, which produces a methylated product from SAM called S-adenosyle-homocysteine (SAH) which can be toxic if accumulated. Therefore, the cell rapidly acts on SAH to produce adenine and S-ribosyl homocysteine (SRH) using the nucleosidase Pfs. While transforming SAH to SRH, Pfs uses a water molecule to produce Adenine. In the next step the LuxS protein catalyses reactions which use SRH to produce both homocysteine and 4,5-dihydroxy-2,3-pentamidine (DPD). The effect of this is to create a feedback loop in the pathway by transforming homocysteine to methionine, and also to rearrange DPD to eventually produce more AI-2 [24].

On another branch of the pathway, SAM decarboxylase converts SAM to Decarboxylated SAM (De-SAM) by releasing *CO*_2_. After that Spermidine synthase transforms De-SAM to 5 ^′^-methylthioadenosine (MTA) using putrescine and producing spermidine. The Pfs enzyme also converts MTA to 5 ^′^-methylthioribose (MTR) while using a water molecule and producing polyamines; MTR is later excreted from *E.coli* [53]. Note that ubiquitous molecules such as *CO*_2_ and water are not represented in our model.

The model has 21 places, and 23 transitions. The Li model has a specific finite number of tokens in each place and specific rate constants, all being derived from experiments [24]. There are two source places, i.e. with only outgoing arcs and thus an always limited number of tokens, reflecting limited environmental resources: *Nutrients* and *Putrescine*. Two other places maintain a constant value: the Pfs and LuxS genes, modelling the fact that genes are not used up by transcription. In summary, the model describes the pathway starting from the sources places to *AI2_Out* which represents the AI-2 produced in Phase 2 excreted to the environment. See Tables 1 and 2 respectively for an explanation of the names of the places and transitions in the Phase 2 model.

**Table 1:** Phase 2 Places

Place name	Biological entity
Adenine	Adenine
AI2_In_Phase2	AI_2 produced in phase 2 in the cell
AI_2_Out	AI_2 exported into the environment
Decarb_SAM	Decarboxylated S-adenosylmethionine
DPD	4,5-dihydroxy-2,3-pentamidine
Homocys	homocysteine
LuxS_gene	LuxS genes
LuxS_mRNA	LuxS mRNA
LuxS_prot	LuxS protein
Met	methionine
MTA	5 ^′^-methylthioadenosine
MTR	5 ^′^-methylthioribose
Nutrients	Nutrition source
pfs_gene	nucleosidase Pfs genes
Pfs_mRNA	nucleosidase Pfs mRNA
Pfs_prot	nucleosidase Pfs protein
Putrescine	putrescine
SAH	S-adenosylehomocysteine
SAM	S-adenosylmethionine
Spermidine	Spermidine
SRH	S-ribosyl homocysteine

**Table 2:** Phase 2 Transitions

Transition name	Biological action
AdoMet	Methioninadenosyl transfer
AI2_excret	AI_2 excretion
AI2_syn	AI_2 synthesis
bio_reac	General bioreaction over nutrients
DPD_deg	DPD degradation
LuxS_mRNA_deg	LuxS mRNA degredation
LuxS_prot_deg	LuxS protein degredation
LuxS_transcription	LuxS transcription
LuxS_translation	LuxS translation
MethyTrans	Methyl transfer
Met_recover	Recovery of Methionine
MTR_excret	Excretion of MTR
MTR_syn	MTR synthesis
Pfs_mRNA_deg	Pfs mRNA degredation
Pfs_prot_deg	Pfs protein degredation
pfs_transcription	Pfs transcription
pfs_translation	Pfs translation
Polyamin_util	Utilisation of Polyamine
SAH_Hydro	SAH Hydrolysis
SAM_Decarb	SAM decaroxylation
SpeE_syn	Spermidine synthesis
Spermi_util	Spermidine utilisation
SRH_cleave	SRH cleavage

In the following, place and reaction names are the short form given in the actual model and indicated in *italic*.

### Phase 3 model – biofilm formation

Our Phase 3 biofilm formation model follows the wording in the papers by Li et al. [26] and Novak et al. [27], neither of which presents a formal model, but rather provide textual descriptions of the various sub-mechanisms. In the following we consider the production of the biochemical components of the biofilm matrix rather than the formation and structural properties of the matrix itself.

In order for the process of biofilm formation to be activated, bacteria actively transport AI-2 into the cell if they detect that the external concentration of the molecule is above some threshold, implying that the population of bacteria is high enough to initiate this pathway as well as other pathways [26]. Then the next stage of quorum sensing starts; AI-2 enters the bacteria in the stationary phase through a transporter complex called LuxS Regulator ABCD (LsrABCD). Thus, when there is insufficient AI-2, the cell will not detect it and biofilm formation will not be initiated [6, 54].

As soon as AI-2 enters the cell, it is phosphorylated by the kinase LsrK. The phosphorylated AI-2 counteracts the repression of the lsr genes by LsrR, by binding to and thus separating the repressor LsrR from the lsr genes [26, 53]. This allows the lsr genes to actively produce the proteins *LsrABCD*, *LsrK* and *LsrR*, all of which are degraded.

The biochemical components of biofilm (carbohydrates etc.) – hereafter called ‘biofilm’ for short – can be formed in two ways; firstly by the interaction between *AI2_In* and *QSeBC* via a complex process represented overall by the transition *BiofilmFormationAI2*. *QSeBC* (Quorum sensing E.coli regulator B and C) is a coupling mechanism that senses and responds to environmental changes in order to control biofilm formation [27]. The alternative route for the formation of biofilm is via *LsrR* which requires the presence of *AI2_In* as well [26], again via a complex process represented overall by the transition *BiofilmFormationLsrR*.

**Fig. 4:**
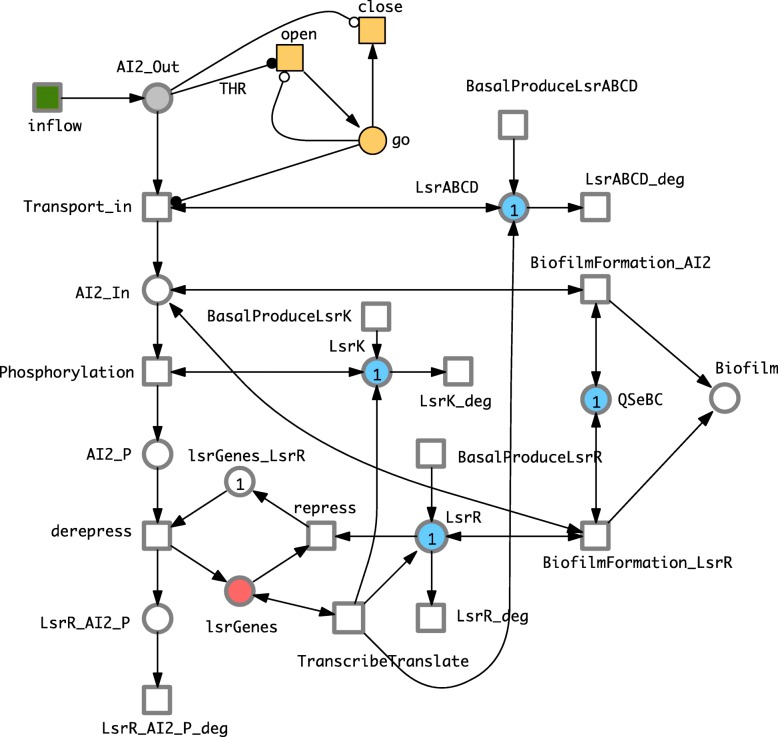
Phase 3 Biofilm production model. To graphically distinguish discrete and continuous nodes, we adopt the usual drawing convention of representing discrete nodes by thin lines, and continuous nodes by thicker lines. The arc weight for the read arc going from go to *Transport_in* is given by the integer constant *THR*. All transition rates follow mass-action kinetics with specific rate constants (not shown); see Additional file 2 for details. Colour code: yellow - discrete nodes (the go mechanism), green - infinite inflow, blue - protein (complexes), red - genes. This model can be equally read as SPN or HPN

See Fig. 4 for a graphical representation of the model, which comprises 11 places and 15 transitions; in addition we have introduced a control mechanism (“Go system”) to ensure that AI-2 will enter the cell only when a certain threshold is reached [54], and also that AI-2 can build up in the environment and consequently diffuse as a signal to other cells in the environment. This models the fact that under low population densities AI-2 will keep diffusing in the environment and never enter the cell, with the consequence that biofilm will never be formed [12].

In order to impose a threshold on the activity of the *Transport_in* transition, we exploit two special arcs available in Extended Petri nets. Special arcs always connect a place to a transition. Read arcs (represented by black circles as arrow head) test if the marking of the pre-place is larger or equal than the arc weight, which we specify by the constant *THR*. Inhibitory arcs (represented by hollow circles as arrow head) test if the marking of the pre-place is smaller than the arc weight; in our case we use (so far) the weight 1, which is usually not shown. Both special arcs influence the enabledness of a transition, but do not change the marking of the tested places upon firing. Combining both as shown in the Fig. 4 (yellow subnet) brings the required control mechanism. As we want to obtain a sharp on/off mechanism, we implement the Go control mechanism as a discrete component. The stochastic transition *open* is enabled if *m*(*AI*2_*Out*)≥*THR*∧*m*(*go*)<1, and the stochastic transition *close* if *m*(*AI*2_*Out*)<1. Obviously, both conditions preclude each other for any *THR*≥1; thus there is always at most one token on the discrete place *go*, which appears as a side-condition for the transition *Transport_in*. Consequently, the rate (following the mass-action pattern) is zero, if *m*(*go*)=0, and else the rate depends only on the other two pre-places (*AI2_Out*, *LsrABCD*).

Due to the stochastic nature of the go mechanism, the Phase 3 model can either be read as an entirely stochastic model, or as a hybrid model keeping the go mechanism stochastic and the remainder being continuous. Moreover, the model implements the repression-derepression transcription cycle of lsr genes which also should be modelled stochastically because gene expression is a fundamentally stochastic process [55]. This suggests that the two places and transitions involved in this cycle can be kept as stochastic in the hybrid model as well.

See Tables 3 and 4 respectively for an explanation of the names of the places and transitions in the Phase 3 model.

**Table 3:** Phase 3 Places

Place name	Biological entity
AI2_In	Imported AI_2
AI2_Out	AI_2 in the environment
AI2_P	Phosphorylated AI_2
Biofilm	Biofilm
go	AI_2 threshold control system
LsrABCD	LsrABCD protein
lsrGenes	lsr genes
lsrGenes_LsrR	The complex of LsrR and lsr genes
LsrK	LsrK protein
LsrR	LsrR protein
LsrR_AI2_P	The complex of LsrR and phosphorylated AI_2
QSeBC	Quorum sensing E.coli regulator B and C

**Table 4:** Phase 3 Transitions

Transition Name	Biological action
BasalProduceLsrABCD	Basal production of LsrABCD proteins
BasalProduceLsrK	Basal production of LsrK protein
BasalProduceLsrR	Basal production of LsrR protein
Biofilmformation_AI2	Biofilm formation via AI_2
Biofilmformation_LsrR	Biofilm formation via LsrR
close	Closure of AI2 import control system
derepress	Derepression of lsr genes
inflow	Inflow of diffused AI2
LsrABCD_deg	Degradation of LsrABCD complex
LsrK_deg	LsrK degradation
LsrR_AI2_P_deg	LsrR_AI2_P degradation
LsrR_deg	LsrR degradation
open	Opening of AI2 import control system
Phosphorylation	AI_2 Phosphorylation
repress	Repression of lsr genes
TranscribeTranslate	Trascription and translation of lsr genes
Transport_in	Importation of AI_2 into the cell

Please note that this is a new model and no precise rates, rate parameters or initial concentrations are available in the literature. We assume mass-action kinetics, and the rate parameters have been uniformly set in three categories, see Table 5 for details. The initial marking comprises four categories, see Table 6 for details.

**Table 5:** Rate parameters for the biofilm formation component

Category	Ratio	Transitions
low	1	basal transcription:
		*BasalProduceLsrABCD*, *BasalProduceLsrK*, *BasalProduceLsrR*;
		degradation: *LsrABCD_deg*, *LsrK_deg*, *LsrR_deg*,*LsrR_AI2_P_deg*;
medium	10	all other transitions
high	10^4^	*open*, *close*

**Table 6:** Initial concentration (marking) for the biofilm formation component

places	comment	initial value
*QSeBC*	a constant place which never changes	1
*lsrGenes_LsrR*	the inhibited state of the lsr genes^1^	1
*LsrABCD*, *LsrK*, *LsrR*	the Lsr proteins	1
else	all other places	0

^1^corresponding to their basal production required to activate the biofilm formation system [66]

### Combining phase 2 & 3 models

**Fig. 5:**
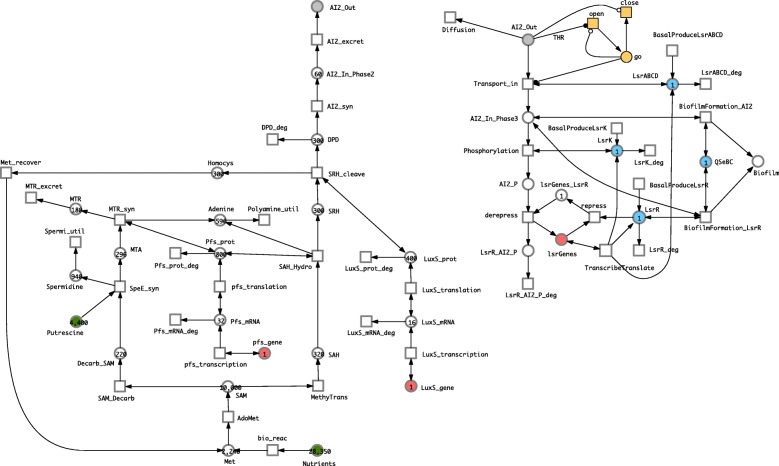
Combined model. The two places *AI2_Out* given in grey are logical places, connecting the two model components, compare Figs. 3 and 4. This model can be equally read as SPN or HPN when keeping the partitioning into discrete and continuous nodes as in Fig. 4

We created an overall model, see Fig. 5, describing AI-2 production and biofilm formation by combining the Phase 2 model based on Li [24] described in “Phase 2 model – AI-2 production” section with the Phase 3 model for production of biofilm described in “Phase 3 model – biofilm formation” section. This was achieved by linking these two models using the place *AI2_Out*, which we represented in each model as a logical place (a shared place appearing in both models). To keep all place names unique, we named *AI2_In* from Phase 2 as *AI2_In_Phase2*, and likewise for *AI2_In* in Phase 3. In preparation for the spatial interpretation of the combined system, we provide the basis of the AI-2 broadcast mechanism. For this purpose we add a sink transition *Diffusion* summarising diffusion to the neighbourhood in any direction; the kinetic constant for diffusion is multiplied by the number of neighbours (2, 8 and 26 in 1D, 2D and 3D, respectively). In this way, the *Diffusion* transition will enable transmission of the broadcast signal via dispersion thus facilitating long-range communication in the spatial model below.

We also adjusted the estimated rates in the Phase 3 model to concord with the general ranges of the specific rates in the Phase 2 model, low being set to 10^−4^, medium to 10^−3^, and high to 1. Note that there are two thresholds, one for open and one for close – for the time being we keep the threshold for close at its default value of 1, while varying that for open.

### Adding space

In this section we reuse the coloured Petri net definitions introduced in “Coloured Petri nets” section to describe diffusion in space, which are summarised in the Additional file 1.

In order to add spatial information regarding both the environment of the bacteria, and the size, position and density (“sparseness”) of the bacterial colony we need to define the environment size over a 1, 2 or 3D grid, and the colony as a smaller grid within this environment. The topology of both the environment and the bacterial colony (or “cluster”) are by default linear in the 1D case, a square in 2D and a cube in 3D. We first export the combined model obtained in the previous section to a coloured Petri net and define all colour-related definition as discussed in “Coloured Petri nets” section. Next we add the following annotations to the coloured model.

- We uniformly assign the colour set Grid3D to all places. Thus, every (coloured) place may hold coloured tokens which are colour triples (x,y,z), the x, y, z components of which are defined over a range from 1 to D1, D2 or D3, respectively.
- All (coloured) places are initialised with markings using the *region* function. This will allocate bacteria to all (x,y,z) positions fulfilling the region criterion as specified by its parameterised call *region(x,y,z,M1,M2,M3,R,S1,S2,S3)*.
- We enhance the definition of the *Diffusion* transition, making it two way in order to model the long-range reception of broadcast AI-2 signals.
- All arcs get assigned the arc expression (x,y,z), with one exception: one arc adjacent to *Diffusion* has to get the triple (a,b,c) to provide the parameters for the neighbourhood function serving as guard, see next item.
- All transitions get assigned as guard the function *region*, with one exception: the transition *Diffusion* gets the guard *neighbour3D26*. This ensures that the combined biofilm model for Phase 2 and Phase 3 will only be generated upon unfolding) for those grid positions as specified by the parameters of the function call *region*, and only *AI2_Out* can diffuse over the whole of the environment grid.

**Fig. 6:**
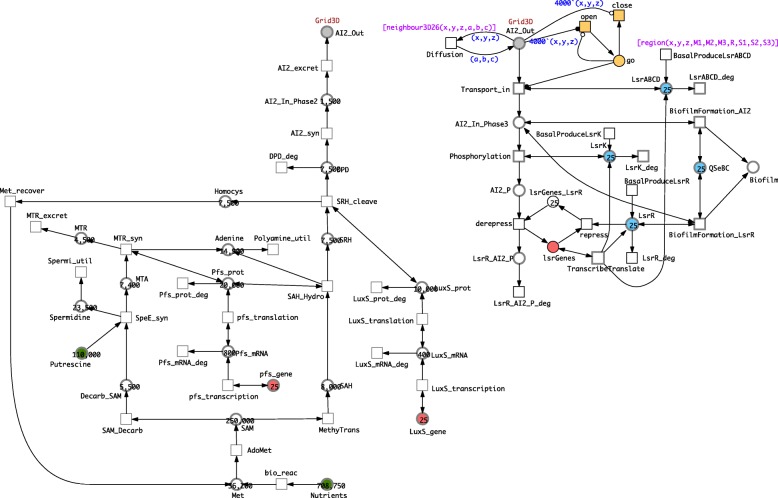
Coloured combined model. The coloured model inherits its structure from the combined model, see Fig. 5, from which it was derived, but enriched by the colour annotations. Typically, uniform annotations are not shown. For illustration, we show them here for a few nodes and arcs, see top right. All other places have the same colour set as the place *AI2_Out*; all transitions for which the guard is not given have the same guard as shown for *BasalProduceLsrABCD*; and all arcs for which the arc expression is not given carry the expression (*x*,*y*,*z*). The initial marking of coloured places shows the total sum of tokens of any colour; e.g., there are 25 coloured tokens on the place *LsrK*, which tells us that the model configuration comprises here 25 bacteria. This model can be equally read as a coloured SPN or a coloured HPN

See Fig. 6 for the resulting model and Additional file 2 for its complete CANDL specification.

## Discussion - model validation

Sound model engineering requires a step-wise model development strategy. Here, we present selected aspects of our step-wise model validation.

### Phase 2 model validation

Note that we consider the Li model as being validated by its publication, but take this opportunity to demonstrate the power of our analysis techniques. We start with standard Petri analysis techniques as known from Petri net theory; see [44] for a summary and related formal definitions in the context of systems biology.

When adding source and sink transitions to model an environment ensuring infinite in/out flow, which makes the Petri net transition-bordered (no sink/source places anymore), the net becomes covered with T-invariants, which is known as a generally criterion for “all transitions may be involved forever in some basic elementary behaviour”.

There are two trivial 1-P-invariants (made of a single place, having always exactly one token), which correspond to the two genes. Due to the added source transitions, the net is structurally unbounded, thus, without timing constraints (i.e. forgetting the rates), all other places (besides those involved in P-invariants) are unbounded.

There are only the two trivial siphons, established by the two 1-P-invariants; consequently, the Siphon Trap Property (STP) holds (because a P-invariant is a siphon and a trap as well). As the net also belongs to the net class Extended Simple (ES), we can conclude that the net is live, i.e. all transitions will forever contribute to the system behaviour. Please note, this conclusion for a structurally unbounded net is possible thanks a structural criterion (STP & ES), and this is true for any timing constraints because it is known that an ES Petri net is time-independently live [56]. See Additional file 3 for more details of these structural analysis results.

We continue with simulative model checking. It is known that the results may depend on the simulation trace, specifically on the step size of the ODE solver and the granularity of the recorded trace, see, e.g., [49, 57]. We chose to simulate the continuous Phase 2 model using the classic Runge-Kutta solver in Snoopy over 10,000 time points and recorded 10,000 observations and truncating the accuracy up to 2 digits after the decimal place. There are 21 species (places), which we categories using the simulative Model Checker MC2 by means of a property library, we developed previously [58]; compare Fig. 7.

1. **always steady state zero:**

**Fig. 7:**
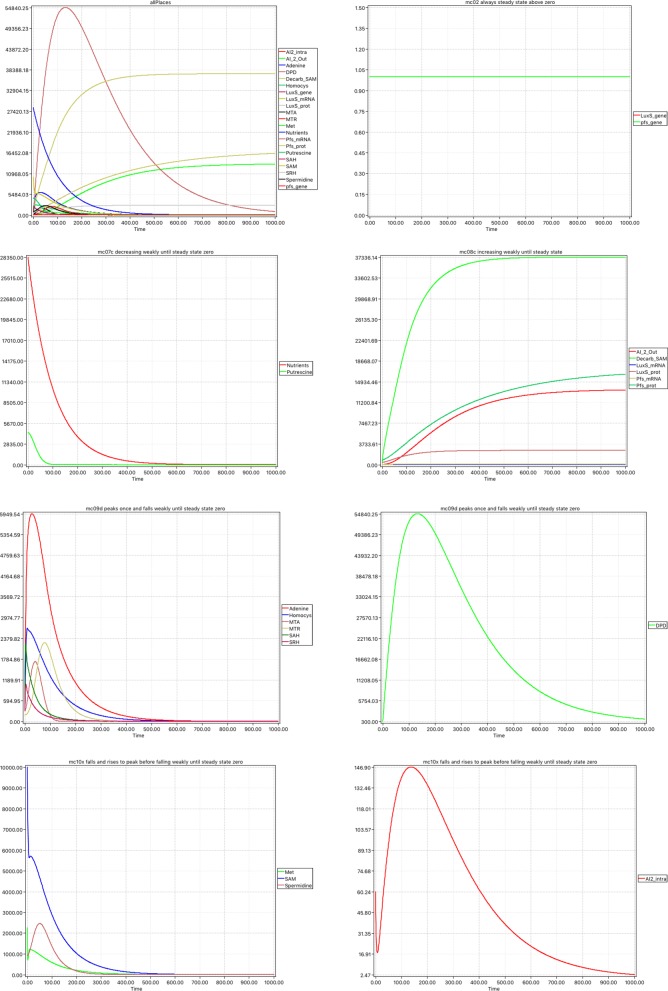
Validating Phase 2 model. Simulation traces for the 5 categories obtained by simulative model checking. The last two rows show in two diagrams each the entities which have been categorised together. For comparison, top left shows all places together. See also Additional file 3: Figure S3
  *P*_≥1_[ *G*([*$x*]=0) ]
  There are no corresponding variables, i.e. there are no entities which are never active.
2. **always steady state above zero:**
  *P*_≥1_[ *G*(*d*[*$x*]=0 & [*$x*]>0) ]
  holds for two entities, which are genes – in this model these are side conditions, they are always active, but never consumed: *luxS_gene* and *pfs_gene*.
3. **decreasing weakly until steady state zero:**
  *P*_≥1_[ *G*(*d*[*$x*]≤0) & ¬*G*(*d*[*$x*]=0) & ((*G*(*d*[ *$x*] ≤0) & ¬*G*(*d*[ *$x*]=0)) *U* (*G*(*d*[ *$x*]=0 & [*$x*]=0))) ]
  holds for the two source places, i.e. places without pre-transitions, which reflect our assumption in the model of finite supply: *Nutrients* and *Putrescine*.
4. **increasing weakly, until steady state:**
  *P*_≥1_[ *G*(*d*[*$x*]≥0) & ¬*G*(*d*[*$x*]=0) & ((*G*(*d*[*$x*]≥0) & ¬*G*(*d*[*$x*]=0)) *U* (*G*(*d*[*$x*]=0))) ]
  holds for six entities comprising the sink place *AI_2_Out*, and two pairs of protein and mRNA which are produced and degraded continuously until production and degradation are balanced *LuxS_mRNA*, *LuxS_prot*, *Pfs_mRNA*, *Pfs_prot*, and also for *Decarb_SAM* where the inflow and outflow eventually become balanced.
5. **peaks once and falls weakly until steady state zero:**
  *P*_≥1_[ *d*[*$x*]>0) & (*d*[*$x*]>0 *U* (*G*(*d*[*$x*])≤0 *U* (*G*(*d*[*$x*]=0 & [*$x*]=0)))) ]
  holds for seven entities: *Adenine*, *DPD*, *Homocys*, *MTA*, *MTR*, *SAH*, and *SRH*.
6. **falls and rises to peak before falling weakly until steady state zero:**
  *P*_≥1_[ ((*d*[*$x*]<0) & (*d*[*$x*]<0 *U* *F*((*d*[*$x*]>0) & (*d*[*$x*]>0 *U* (*F*((*d*[*$x*]<0) & (*d*[*$x*]<0 *U* (*G*(*d*[*$x*]=0) & [*$x*]=0)))))))) ]
  holds for four entities: *AI2_intra*, *Met*, *SAM*, and *Spermidine*.

### Phase 3 model validation

Standard Petri net analysis techniques do not cover inhibitory arcs; so we preclude the go mechanism from the structural analysis. Applying the same approach as we did for the first component relating to Phase 2 confirms again that the transition-bordered version of the Petri net is covered with T-invariants, and reveals two P-invariants - a trivial one, comprising *QSeBC*, and another one comprising the two states (repressed, de-repressed) of the lsr genes, i.e., the places *lsrGenes_LsrR* and *lsrGenes*. Moreover, the STP holds; but because the net structure is beyond ES we can only structurally deduce that the Petri net is free of dead states; see Additional file 3 for details.

Next, we considered our model under two conditions: (i) unlimited amount of *AI_2_Out* and (ii) limited amount of *AI2_Out*. Each condition was simulated in the stochastic setting using the Gillespie [31] algorithm. The simulation was also performed in the hybrid setting using a simulator comprising three components: (1) an ODE solver, we specifically use the CVODE library [59], for the continuous part, (2) Gillespie simulation for the stochastic part, and (3) the synchronisation between the continuous and stochastic net components is done via the improved Hybrid Rejection-based Stochastic Simulation Algorithm (HRSSA) [60], which combines the accelerated method introduced in [60] with the hybrid rejection-based stochastic algorithm from [61].

**Fig. 8:**
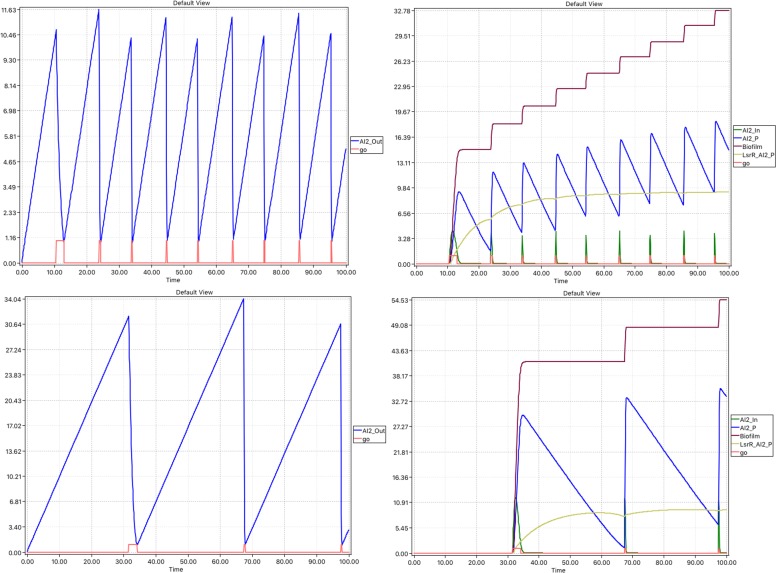
Phase 3 model (hybrid) – unlimited supply. Top row: THR=10, bottom row: THR=30. Left column: go opens when AI2_Out exceeds the THR up to some stochasticity; Right column: internal mechanism driving stepped accumulation of Biofilm

Under the first condition we observed a repeatedly peaking behaviour for most of the metabolites because the *go* place is repeatedly opening and closing. These metabolites were *AI_2_In*, *AI_2_Out*, *AI_2_P*, *LsrABCD*, *LsrK*, *LsrR_AI_2_P*, *lsrGenes*, *lsrGenes_LsrR* and *go*. Because of its accumulatory nature, *Biofilm* increases stepwise. See Fig. 8 for the hybrid results The stochastic results are not reported as they are similar for a sufficiently high number of runs.

In contrast, with a limited amount of *AI_2_Out* no repeated peaks were observed for most of the places because the *go* place is immediately occupied once and then becomes empty later on.

**Fig. 9:**
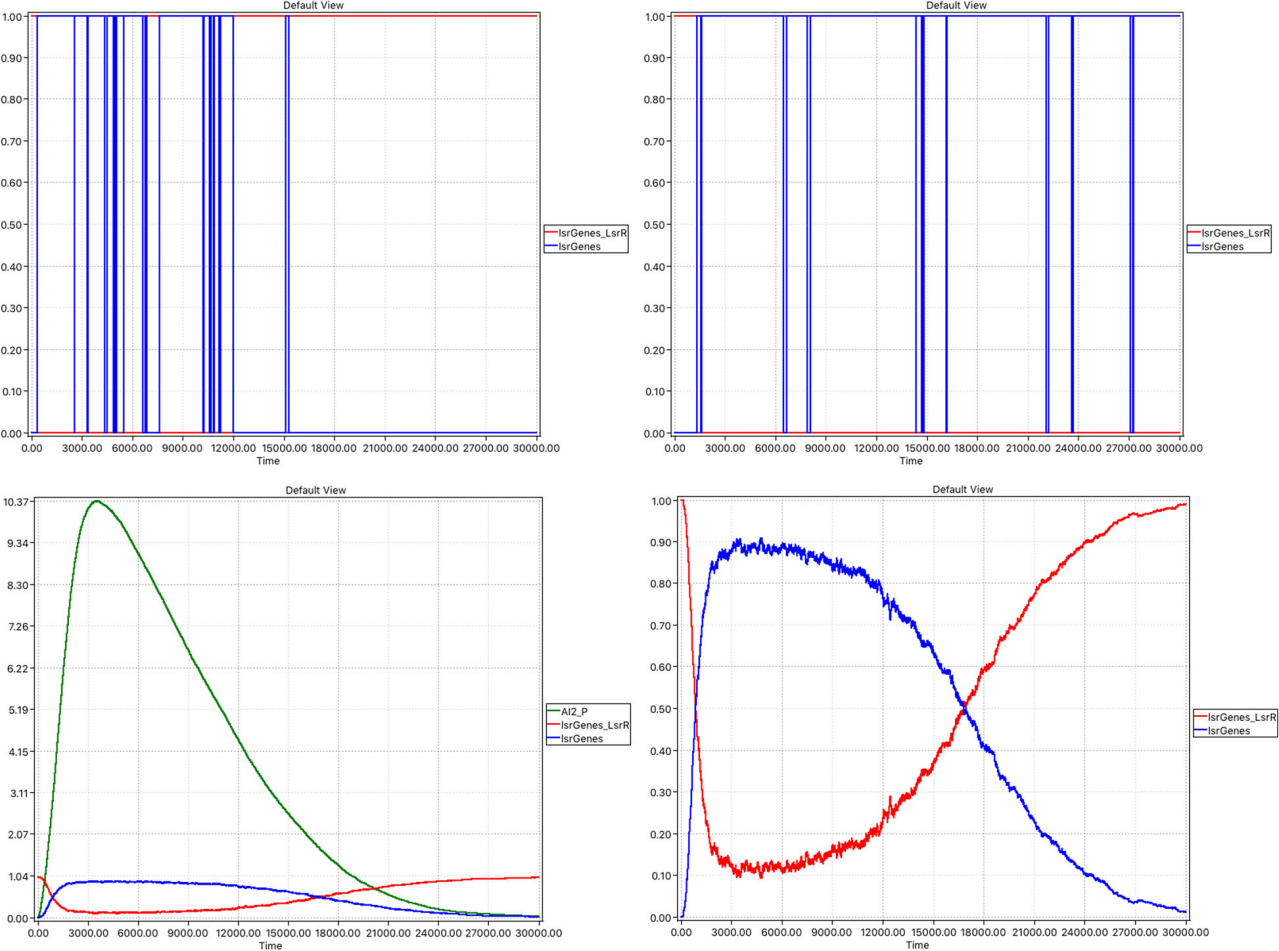
Phase 3 model (stochastic): limited supply. Focus on repression–derepression machinery causing changes between states with lsrGenes bound or unbound to LsrR. Top row: two single runs illustrating stochasticity of the repression–derepression machinery; Bottom row: average over 1000 runs of repression–derepression machinery showing initial repressed state will be reached again as AI2_P approaches zero steady state

To explore the repression–depression machinery causing changes between the states of *lsrGenes* bound or unbound to *LsrR*, we performed stochastic runs in the limited *AI2_out* scenario. The on-off nature of the binding is clearly observable in single runs, whereas the nature of the overall state switch driven by the availability of *AI2_P* is apparant in the average of many stochastic runs, see Fig. 9. The average behaviour can equally be observed in the hybrid setting. The initial repressed state will be reached again as *AI2_P* approaches zero steady state; this is not observed in the unlimited *AI_2_Out* setting because *AI2_P* never approaches a zero steady state.

### Validation of the non-spatial combined model

We start with the following observations of some specific aspects of the model behaviour. Increasing the threshold *THR* of the transition *open* (in the following briefly called *THR*_*open*) postpones the inflow into the biofilm component and allows higher values of *AI2_Out* to accumulate. When *THR* of the transition *close* (in the following briefly called *THR*_*close*) is close to 1, i.e. the gate rarely closes, then it takes longer until the *AI2_In_Phase3* is processed by the biofilm component, because there is an higher inflow.

When *THR*_*close* is close to *THR*_*open* (*THR*_*close*≤*THR*_*open*), then the thresholds strongly influence how much of the AI-2 is used by the biofilm component and how much is left to diffuse in space by the transition *Diffusion*. The experimental evidence [62] is that the biological threshold mechanism is symmetrical in that the opening and closing of the AI2 inward transport channel occur at the same threshold value, which we have reflected by making the values for open and close identical.

**Fig. 10:**
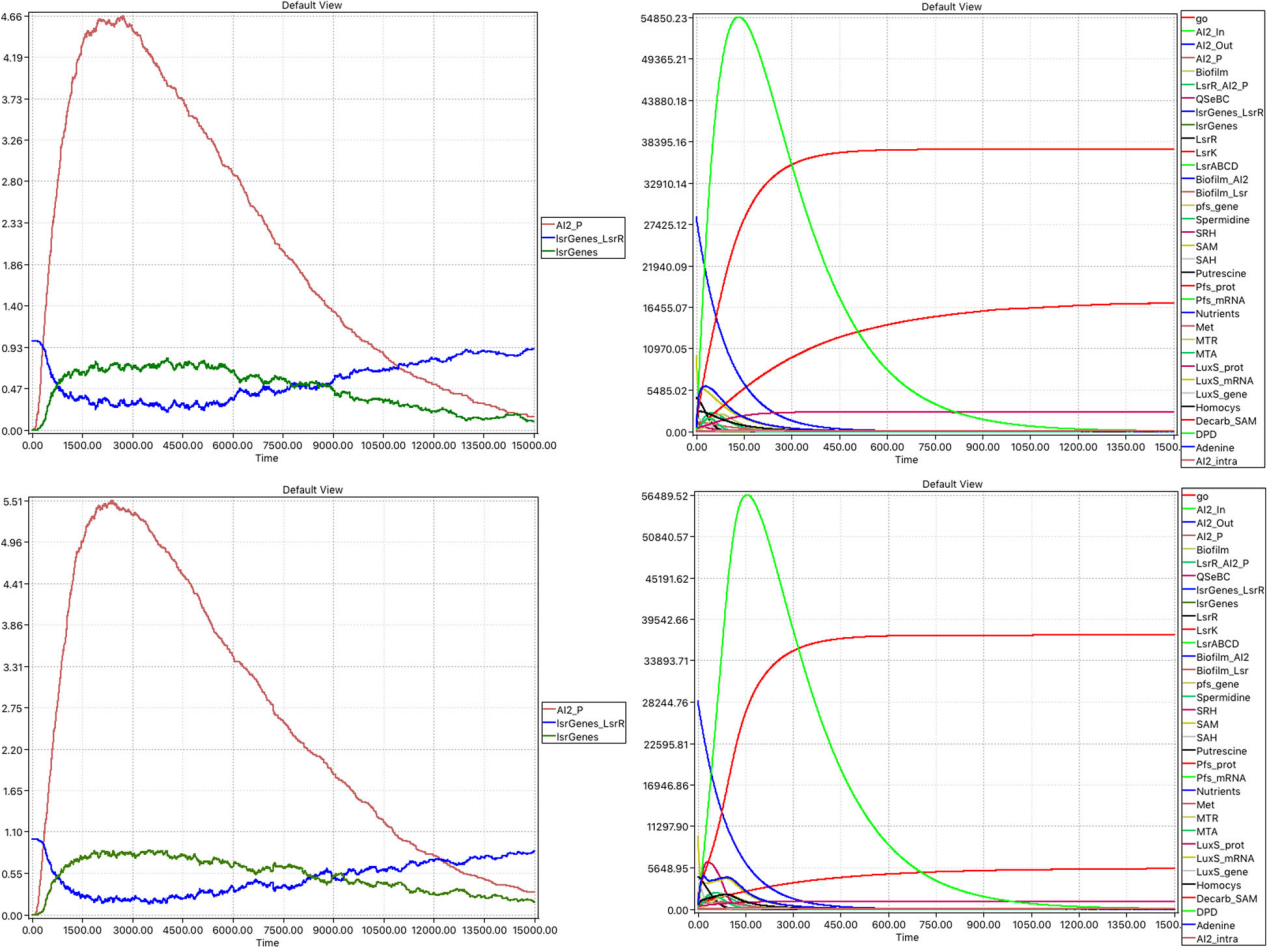
Combined non-spatial model (stochastic). Average over 100 runs of (left) repression–derepression machinery showing initial repressed state will be reached again as AI2_P approaches zero steady state, (right) all species. First row: Gillespie, second row: Delta leaping

We found that the lsr genes eventually reach the repressed state in the steady state using the following values: diffusion parameter *kd*=0.1,*THR*_*open*=*THR*_*close*=40, with a simulation time = 15000, see top row in Fig. 10, and compare with Fig. 9. While performing these experiments we noticed that the runtimes to simulate the hybrid model were considerably greater than those for the stochastic simulation (using Gillespie: 95s, hybrid: 3609s), caused by increasing stiffness and discontinuities which are problematic for continuous solvers. Thus in the following we use stochastic simulations. We further reduced the runtime required for stochastic simulation to 13 s, i.e. by a factor of over 7, by employing the approximative delta-leaping algorithm [32, 63]. Although delta-leaping is an approximative approach, the results are sufficiently close to the exact Gillespie algorithm, see second row in Fig. 10.

Furthermore the behaviour of the combined model can be characterised by a combination of the validation results reported in “Phase 2 model validation” and “Phase 3 model – biofilm formation” sections, which reflects the fact that the two components have a simple interface comprising just one species (place).

### Validation of the spatial combined model

The validation is carried out by simulating an automatically unfolded stochastic Petri net. The size of the unfolded net obviously depends on the size of the grid, i.e., its resolution, and on the number of bacteria we position on the grid (the exact position does not matter). Due to the regular structure of our coloured model, we are in the favourite position to be able to provide closed formulae specifying the size of the underlying uncoloured model. The coloured Petri net comprises 34 places and 40 transitions. Thus, with *N* being the number of bacteria and *D* the size of the square grid, we obtain by unfolding (33*N*+*D*^2^) places and (39*N*+8*D*^2^−12*D*+4) transitions. For example, with *D*=21 and *N*=25, we obtain an unfolded Petri net comprising 1266 places and 4255 transitions, and with *D*=101 and *N*=25 we obtain 11,026 places and 81,375 transitions.

We explored the behaviour of the model for different configurations: a single bacterium, and for colonies with 25 bacteria under different sparseness conditions, in two different environment grids (21x21 and 101x101). We simulated each configuration with the stochastic approximative Delta leaping solver for the same time period (100 runs for 20,000 time steps and recording 1000 time points).

In all cases the *AI2_Out* peaks before the Biofilm reaches a steady state due to the exhaustion of *Nutrients* and *Putrescine* in the Phase 2 part of the model.

We found that the single bacterium produced an extremely small amount of biofilm. In all the other configurations, although the production of *AI2_Out* is the same in all these cases because the number of bacteria is the same, the total amount of *AI2_Out* remaining in the environment increased as the sparseness increased, because less is absorbed by the colony under sparse conditions.

**Fig. 11:**
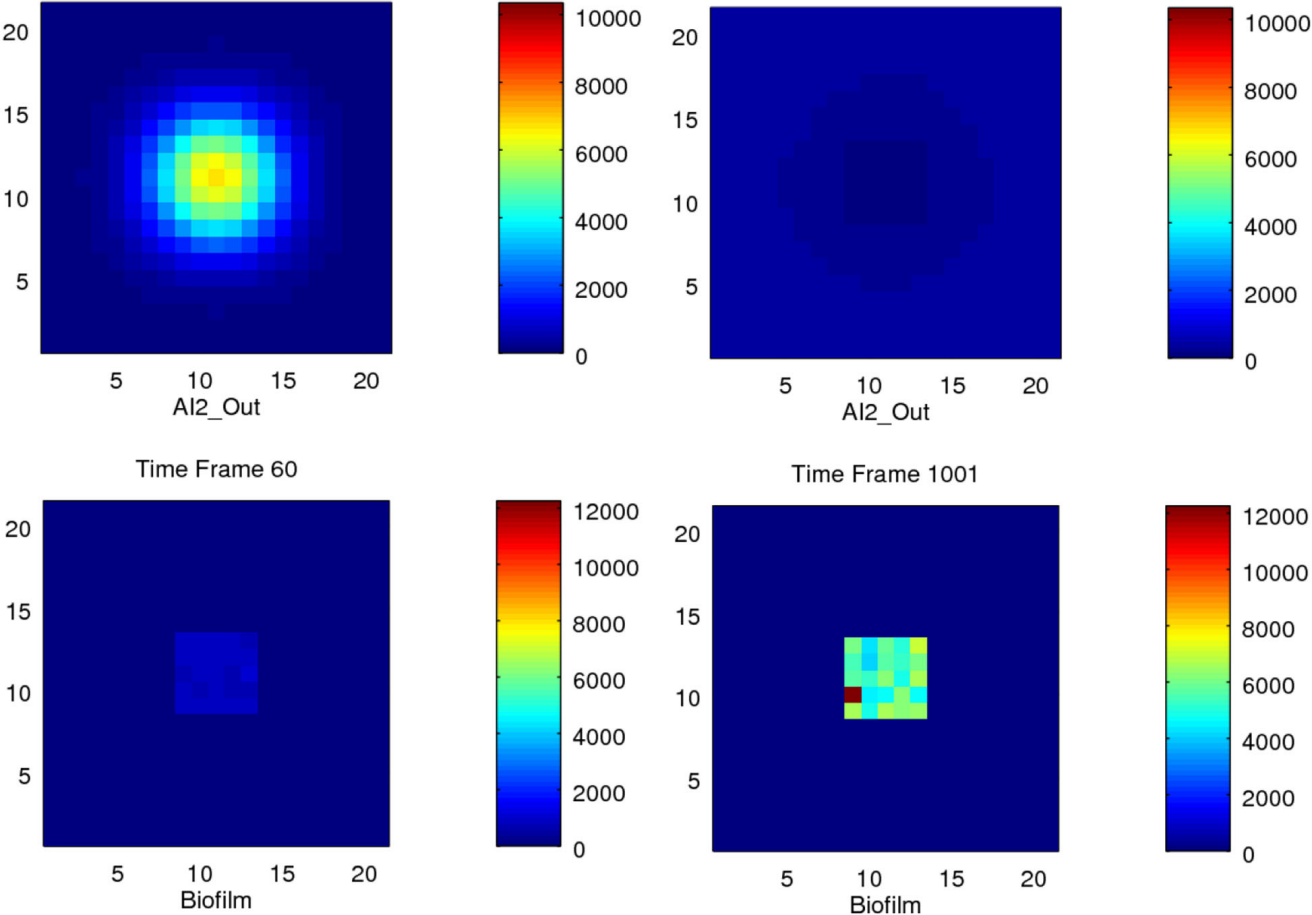
Stochastic simulation of a dense colony. Heatmap visualisation for 25 bacteria in a dense colony in a 21x21 grid size environment, showing two time points

**Fig. 12:**
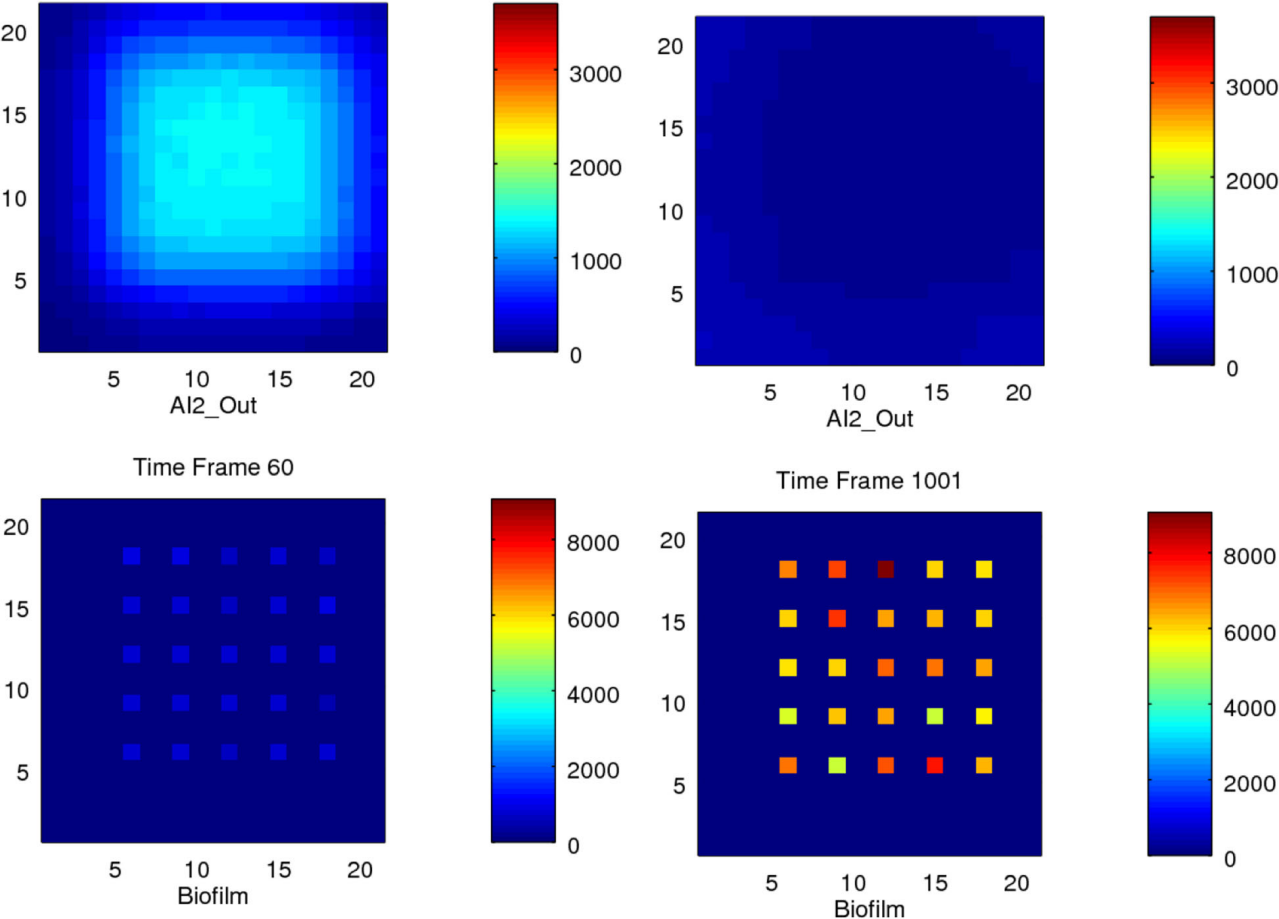
Stochastic simulation of a sparse colony. Heatmap visualisation for 25 bacteria in a sparse colony in a 21x21 grid size environment, showing two time points

Further, we observed that the total amount of Biofilm noticeably decreased with sparseness, because *AI2_Out* diffuses more rapidly away from the bacteria in sparse situations, thus reducing the signal required to trigger the threshold and maintain the production of biofilm; see Tables 7 and 8. We also found that that the values of biofilm at the central positions of a compact colony are higher than those at the edges, see Figs. 11 and 12.

**Table 7:** Total values of AI2_Out and Biofilm, for the 21x21 colony

Colony	AI2_Out	Biofilm
(i) R0	13,374.49	0.68
(ii) R2	308,478.60	55,468.61
(iii) R5	326,647.00	637.79
(iv) R7	341,143.57	35.14

(i) a single bacterium, (ii) a compact (non-sparse) colony of 25 bacteria in a 5x5 square, and sparse colonies with (iii) 24 bacteria regularly positioned in a 11x11 square, and 25 bacteria regularly positioned in a (iv) 15x15 square

**Table 8:** Total values of AI2_Out and Biofilm, for the 101x101 colony

Colony	AI2_Out	Biofilm
(i) R0	12966.33	0.8
(ii) R2	302,573.88	51,995.51
(iii) R4	331,666.27	4,023.54
(iv) R7	333,870.79	88.35
(v) R9	334,316.30	29.99
(vi) R11	333,968.73	27.05

(i) a single bacterium, (ii) a compact (non-sparse) colony of 25 bacteria in a 5x5 square, where all all bacteria are adjacent to each other, sparse colonies with 25 bacteria regularly positioned in a (iii) 9x9 square, (iv) 15x15 square, (v) 19x19 square, and (vi) 23x23 square

These observations were in concord with our expectations; so we consider the model as validated. To illustrate the spatial behaviour, we provide movies as supplementary material showing the behaviour of *AI2_Out* and *Biofilm* over time and space, see Additional file 1 for download instructions.

We conclude our model validation with questioning our stochastic simulation strategy by comparing the results produced by the exact Gillespie algorithm with those generated by the Delta-leaping algorithm, for 10 and 100 runs each. For doing this, we generated four traces of simulation length 20,000 recorded at 1,000 time points for a grid square of size 9 in the 2D scenario, hosting a sparse colony of 9 bacteria distributed over a region with a radius of 2. We use the following formula to calculate the absolute difference between two simulation traces over time:

$${AD}(t)=\left|({DataSet1}(t)-{DataSet2}(t))\right| $$

**Fig. 13:**
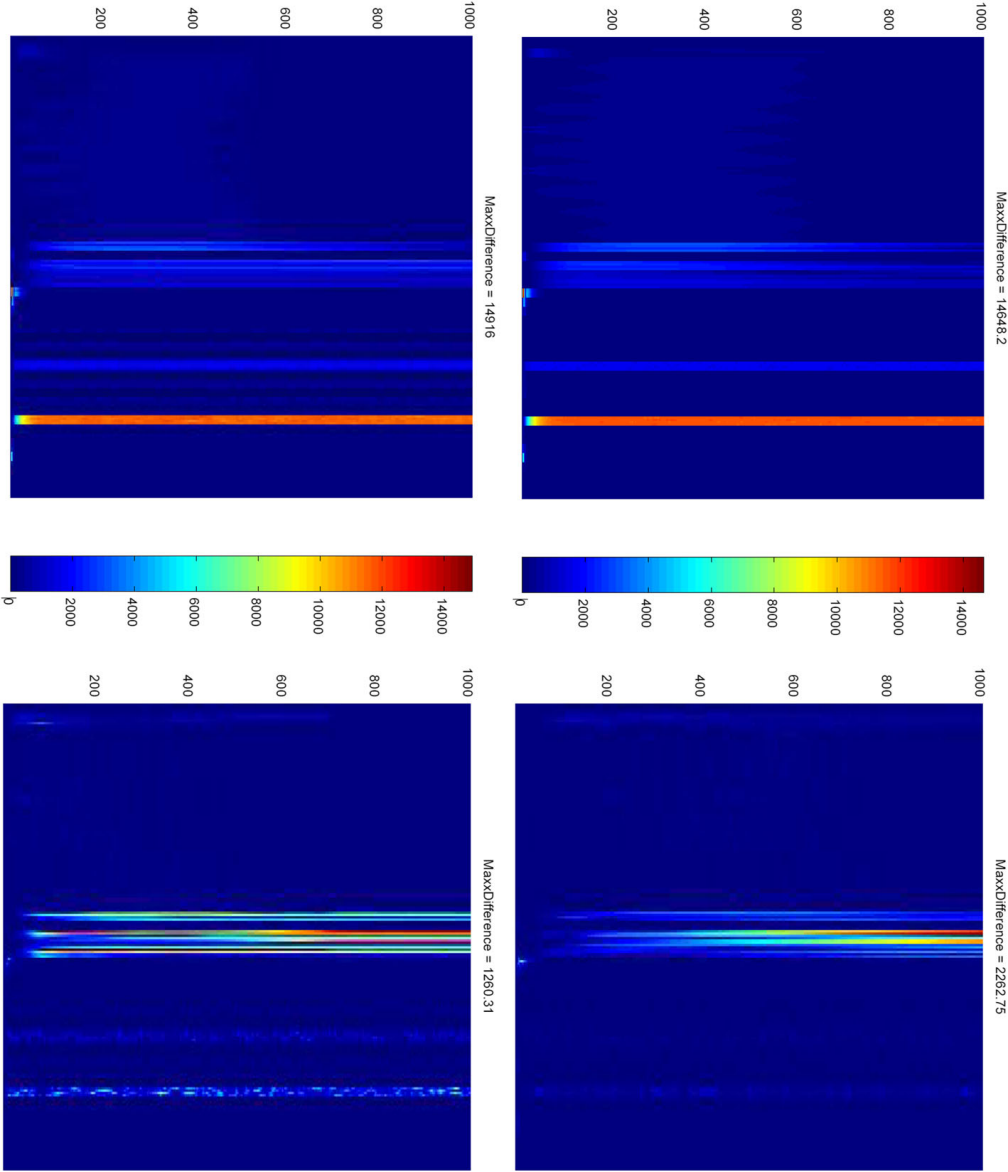
Comparing stochastic simulations. First row: comparing Gillespie with delta leaping, (left) 10 runs, (right) 100 runs. Second row: (left) comparing Gillespie, 10 runs with Gillespie, 100 runs; (right) likewise for delta leaping

The result is represented as a heat map, showing the propagation of the absolute difference encoded by colour over the given time period, where time and the trace variables are represented by x and y axes, respectively; see Fig. 13.

The stripes in the middle relate to the biofilm produced by the 25 bacteria positioned in the middle of the grid, the two thinner stripes in the lower half relate to the two genes in the Phase 2 model, which both have lower token numbers, thus stochasticity has an higher effect. Both stripes can be explained by the stochastic effect not levelled out by the small number of runs. We take the close resemblance of the two heat maps in the first row as a confirmation that the delta leaping simulation method sufficiently well approximates the exact stochastic method, while substantially reducing the runtime, as previously reported [32, 58]. All heat maps together illustrate that there is not much difference between 10 and 100 runs, which we take as a justification not to increase the number of runs over which we average.

## Conclusions

The spatial model which we have developed and presented in this paper describes *E.coli* biofilm formation driven by the Autoinducer 2 (AI-2). We have incorporated the non-spatial model of AI-2 production in Phase 2 of bacterial growth previously published by Li et al. [24], and linked it with our description of biofilm formation in Phase 3. We have described the method by which we have rigorously developed and tested our model in a step-wise manner covering continuous, stochastic, hybrid, and spatial aspects at different levels of abstraction. The spatial representation is achieved using coloured stochastic and coloured hybrid Petri nets, and the behaviour of this version reveals emergent properties not evident in the non-spatial one. For example, we have investigated the model under different configurations ranging from a single bacterium, to colonies under different conditions of sparseness, all contained within environments of different sizes. The model can also be used to investigate the behaviour of models under different host environments, for example finite resources versus those renewed under steady or even cyclical regimes – as occurs in the gut or in oral biofilm. Our results confirm that our model behaves as expected, i.e. that biofilm formation is increased in areas of higher bacterial density. Our research into related work has shown that there is a lack of quantitative experimental data on the effect of population density on quorum sensing driven biofilm formation, and we suggest that this is an open area for exploration by experimentalists.

Although we have explored configurations in 2D in order to keep computations within reasonable time bounds, our approach to encode space is highly flexible, and easily configured for the 1D, 2D or 3D scenario. Our spatial modelling strategy can be equally applied to other problems evolving in time and space.

### Reproducibility

We provide all models in their source format, and use only publicly available tools. Modelling and simulation was done with Snoopy [42]. Additionally, we used Marcie [64] and MC2 [49] for simulative model checking. The 2D visualisation was done by help of Octave [65]; all our scripts are available on request.

## Additional files

Additional file 1 Colouring space. A brief primer illustrating how to deal with coloured Petri nets in Snoopy by means of diffusion in 3D. (PDF 567 KB)

Additional file 2 Complete CANDL specification of the case study. The complete listing of the CANDL specification for the coloured SPN given in Fig. 6. (PDF 184 KB)

Additional file 3 Supplementary material for model validation. Additional explanations and figures illustrating various aspects of the model validation performed. (PDF 3614 KB)

## Abbreviations

1D/2D/3D space: One/two/three-dimensional space
AI: Autoinducer
AI-2: Autoinducer 2
CANDL: Coloured abstract net description language
CPN: Continuous Petri nets
ES net: Extended simple net
HPN: Hybrid Petri nets
IBD: Inflammatory bowel disease
IBS: Irritable bowel syndrome
LTL: Linear-time temporal logic
ODEs: Ordinary differential equations
PDEs: Partial differential equations
PLTLc: Probabilistic linear-time temporal logic with numerical constraints
QPN: Qualitative Petri nets
SBML: Systems biology markup language
SPN: Stochastic Petri nets
STP: Siphon trap property
