# Supplementary material for: Spatial quorum sensing modelling using coloured hybrid Petri nets and simulative model checking
Source: BMC Bioinformatics. 2019 Apr 18;20(Suppl 4):173. doi: 10.1186/s12859-019-2690-z (PMC6471779; doi:10.1186/s12859-019-2690-z)
Supplement: Supplementary file 1 — Colouring space. A brief primer illustrating how to deal with coloured Petri nets in Snoopy by means of diffusion in 3D. (PDF 567 KB) [file 12859_2019_2690_MOESM1_ESM.pdf]

Additional file 1 – Colouring space

This document provides supplementary material for

- *D Gilbert, M Heiner, L Ghanbar, J Chodak: Spatial quorum sensing modelling using coloured hybrid Petri nets and simulative model checking; BMC Bioinformatics, Supplement issue: 12859-20-S4, DOI: 10.1186/s12859-019-2690-z*

The source files described in this Primer can be downloaded from

- *http://www-dssz.informatik.tu-cottbus.de/DSSZ/Software/Examples*

The software tools required are available at

- *http://www-dssz.informatik.tu-cottbus.de/DSSZ/Software/Software*

In our toolkit, a coloured Petri net can either be specified in a graphical way using Snoopy, see Figure 1, or in a textual notation, for which we use the Coloured Abstract Net Description Language (CANDL), see the listing given below. CANDL files can be written with any (plain) text editor, or generated out of a graphical specification by help of Snoopy. CANDL files are read by Snoopy and Marcie.

For more details, please consult manuals and websites of our PetriNuts toolbox:

Manual coloured Petri nets F Liu, M Heiner and C Rohr: Manual for Colored Petri Nets in Snoopy; Technical report 02-12, Brandenburg University of Technology Cottbus, Department of Computer Science, March 2012. http://www-dssz.informatik.tu-cottbus.de/publications/btu-reports/Manual_for_colored_Petri_nets_2012_03.pdf

Manual Marcie M Schwarick, C Rohr and M Heiner: Marcie Manual; Technical report 02-16, Brandenburg University of Technology Cottbus, Department of Computer Science, December 2016. https://opus4.kobv.de/opus4-btu/frontdoor/index/index/docId/4056

Manual coloured hybrid Petri Nets M Herajy, F Liu, C Rohr and M Heiner: Coloured Hybrid Petri Nets in Snoopy - User Manual; Technical report 01-17, Brandenburg University of Technology Cottbus, Department of Computer Science, March 2017. https://opus4.kobv.de/opus4-btu/frontdoor/index/index/docId/4157

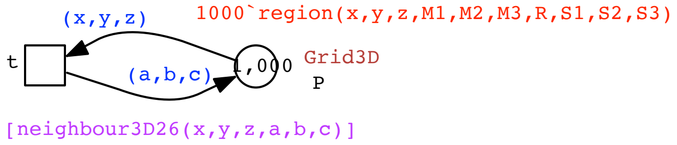

Figure 1 Graphical representation of a coloured Petri net for diffusion in 3D. The initial marking sets 1000 tokens in the centre of the grid, here specified by help of the function *region*. The transition rate (not shown) follows the mass-action law with the kinetic parameter $k = 0.1$. The graphics does not reveal all details; e.g., the definitions of colour sets (here *Grid3D*) and colour functions (here *region*, *neighbour3D26*) are not shown.

The following listing is the complete CANDL specification of the coloured Petri net shown in Figure 1. It can be easily adjusted to a 1D, 2D or 3D scenario.

This coloured Petri net modelling diffusion in 3D provides all colour-related definitions used in our case study of spatial quorum sensing developed in the manuscript. By exchanging the keyword **colspn** with **colcpn** or **colhpn**, the net will be read as a coloured CPN or coloured HPN, respectively.

```
colspn [diffusion_3D26] {

constants:
all:
  int D  = 5;
  int D1 = D;        // grid size of 1st dimension
  int D2 = D;        // grid size of 2nd dimension
  int D3 = D;        // grid size of 3rd dimension, 1 makes it 2D
  int M1 = D1/2+1;   // middle position of 1st dimension
  int M2 = D2/2+1;   // middle position of 2nd dimension
  int M3 = D3/2+1;   // middle position of 3rd dimension
  int R  = 0;        // radius of a (square) region
  int S1 = 1;        // spacing in 1st dimension
  int S2 = 1;        // spacing in 2nd dimension
  int S3 = 1;        // spacing in 3rd dimension

param:
  double k = 0.1;

colorsets:
  CD1 = {1..D1};     // index of 1st dimension
  CD2 = {1..D2};     // index of 2nd dimension
  CD3 = {1..D3};     // index of 3rd dimension
  Distance = {1..D};
  Grid3D = PROD(CD1,CD2,CD3);   // 3D grid

variables:
  CD1 : a; CD2 : b; CD3 : c;
  CD1 : x; CD2 : y; CD3 : z;
  CD1 :x0; CD2 :y0; CD3 :z0;

colorfunctions:

// Is (a,b,c) one of the 26 neighbours of (x,y,z) ?
bool neighbour3D26(CD1 a,CD2 b,CD3 c,CD1 x,CD2 y,CD3 z) {
  (a=x-1 | a=x | a=x+1) & (b=y-1 | b=y | b=y+1)
   & (c=z-1 | c=z | c=z+1) & (!(a=x & b=y & c=z))
   & (1<=x & x<=D1) & (1<=y & y<=D2) & (1<=z & z<=D3) };
```

```
// Is (a,b,c) one of the 6 neighbours of (x,y,z) ?
bool neighbour3D6(CD1 a,CD2 b,CD3 c,CD1 x,CD2 y,CD3 z) {
  (a=x-1 | a=x | a=x+1) & (b=y-1 | b=y | b=y+1)
   & (c=z-1 | c=z | c=z+1)      & (!(a=x & b=y & c=z))
   & (( a=x & b=y) | (b=y & c=z) | (a=x & c=z))
   & (1<=x & x<=D1) & (1<=y & y<=D2) & (1<=z & z<=D3) };

// Is (x,y,z) within the region around the centre (x0,y0,z0)
// with a max distance from the centre of 'radius', and
// a space of xd, yd, zd in between in the x/y/z-axis, resp.
bool region(CD1 x,CD2 y,CD3 z,CD1 x0,CD2 y0,CD3 z0,
        Distance radius,Distance xd,Distance yd,Distance zd) {
  (x0-radius)<=x & x<=(x0+radius)
   & (y0-radius)<=y & y<=(y0+radius)
   & (z0-radius)<=z & z<=(z0+radius)
   & (1<=x & x<=D1) & (1<=y & y<=D2) & (1<=z & z<=D3)
   & (x%xd=0 & y%yd=0 & z%zd=0) };

places:
// region generates a token for all colours (x,y,z)
// fulfilling the region criterion;
// to read this file with Snoopy, write:
// Grid3D P = 1`region(x,y,z,M1,M2,1,R,S1,S2,S3);
// for Marcie + andl converter, write:
Grid3D P = [region(x,y,z,M1,M2,M3,R,S1,S2,S3)]1`(x,y,z);

// adjust the guard to change the neighbourhood relation
transitions:
  t {neighbour3D26(x,y,z,a,b,c)}
     :
     : [P + {(a,b,c)}] & [P - {(x,y,z)}]
     : MassAction(k)
     ;

} // end colspn [diffusion_3D26]
```

The following figures demonstrate different use cases for *diffusion_3D26*:

- Figures 2–4 – three examples of the underlying (plain) Petri net structure automatically generated by unfolding the coloured Petri net, to be read either as SPN, CPN, or HPN;
- Figure 5 – some examples demonstrating the flexibility of the function *region* to specify various types of initial markings (isolated bacterium, dense clump, sparse clump);

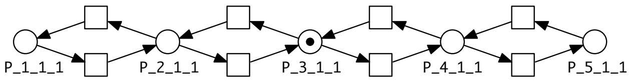

Figure 2 Diffusion in 1D. Generated Petri net for $neighbour3D6$ or $neighbour3D26$ (both yield here the same result), and the constants $D1 = 5, D2 = D3 = 1, R = 0, S1 = S2 = S3 = 1$.

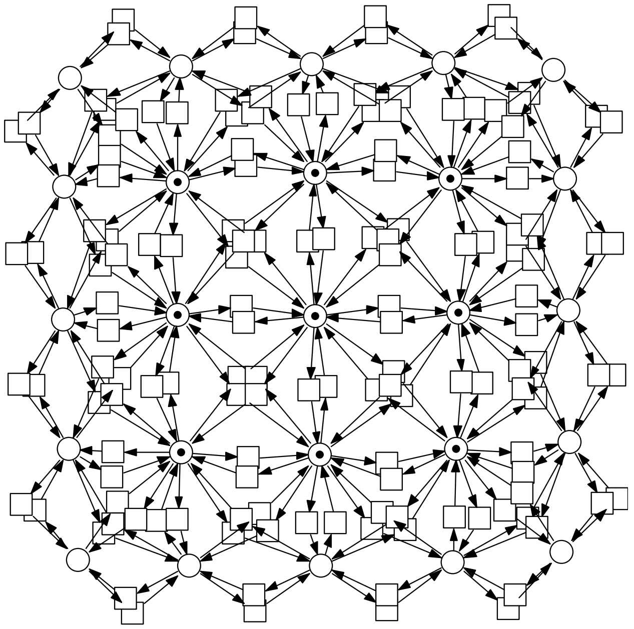

Figure 3 Diffusion in 2D. Generated Petri net for $neighbour3D26$, and the constants $D1 = D2 = 5, D3 = 1, R = 1, S1 = S2 = S3 = 1$.

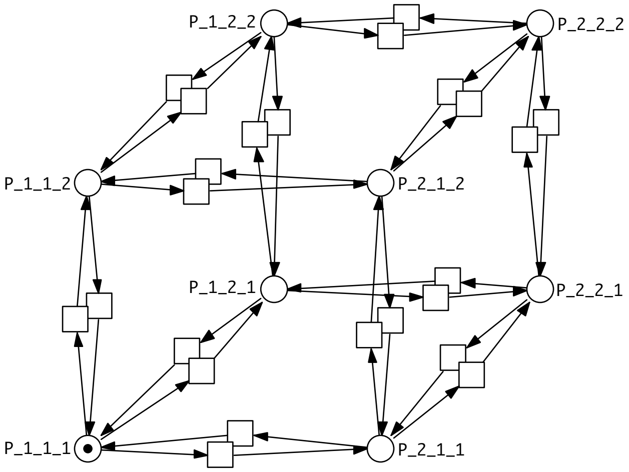

Figure 4 Diffusion in 3D. Generated Petri net for $neighbour3D6$, and the constants $D1 = D2 = D3 = 2, R = 0, S1 = S2 = S3 = 1$.

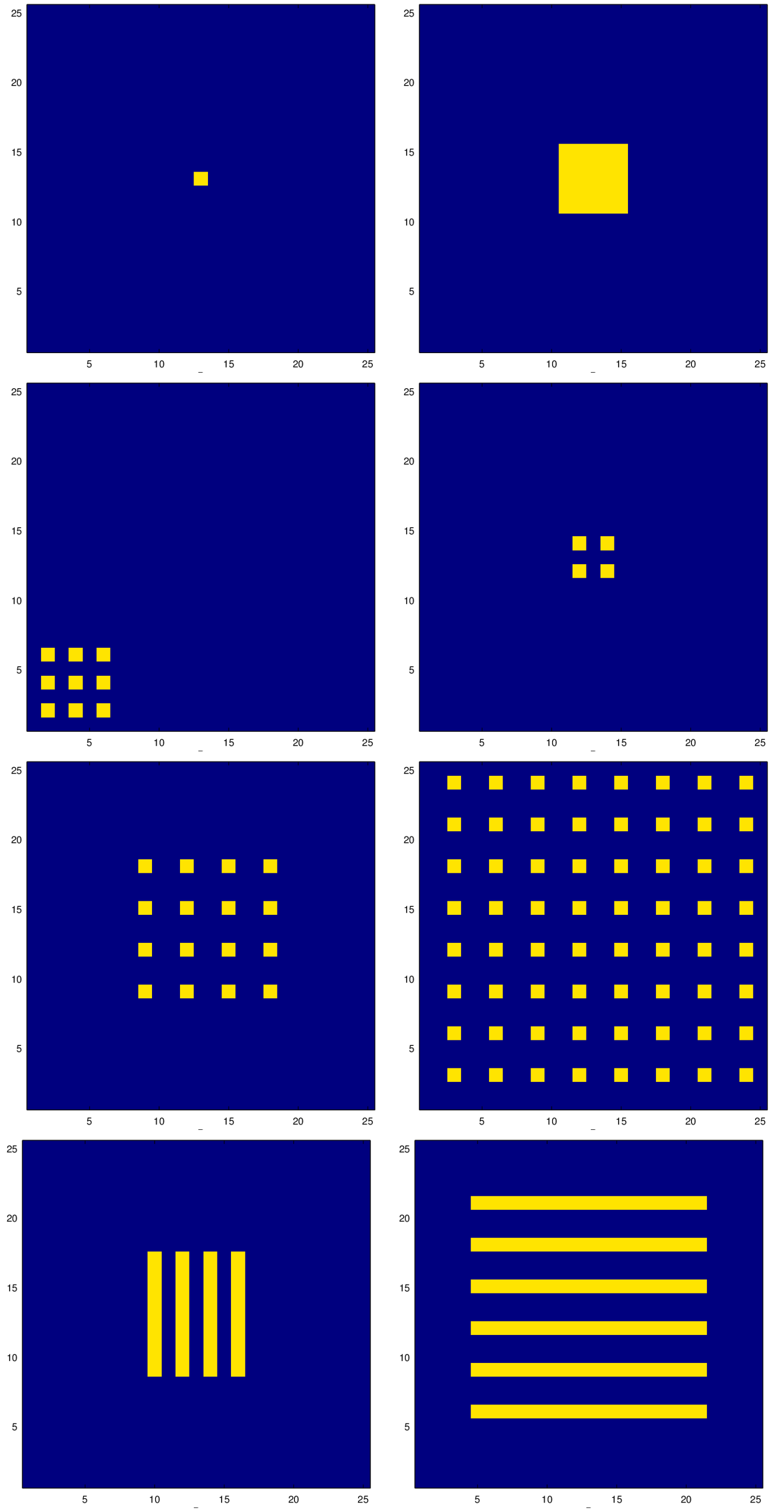

Figure 5 Setting the initial marking. Some examples for the result of the function *region* for different parameter settings in a 2D grid, with D=25. **(a)** isolated mass in the grid's centre position, $R = 0, S1 = S2 = S3 = 1$, see also Figure 2; **(b)** dense clump in the grid centre (25 bacteria), $R = 2, S1 = S2 = S3 = 1$; **(c)** sparse clump in the corner, $R = 3, S1 = S2 = 2, S3 = 1$; **(d)** sparse clump in the grid centre, $R = 2, S1 = S2 = 2, S3 = 1$; **(e)** sparse clump in the grid centre, $R = 5, S1 = S2 = 3, S3 = 1$; **(f)** sparsely covered grid, $R = 12, S1 = S2 = 3, S3 = 1$; **(g)** vertical stripes, $R = 4, S1 = 1, S2 = 2, S3 = 1$; **(h)** horizontal stripes, $R = 8, S1 = 3, S2 = 1, S3 = 1$.
