# Supplementary material for: Spatial quorum sensing modelling using coloured hybrid Petri nets and simulative model checking
Source: BMC Bioinformatics. 2019 Apr 18;20(Suppl 4):173. doi: 10.1186/s12859-019-2690-z (PMC6471779; doi:10.1186/s12859-019-2690-z)
Supplement: Supplementary file 2 — Complete CANDL specification of the case study. The complete listing of the CANDL specification for the coloured SPN given in Fig. 6. (PDF 184 KB) [file 12859_2019_2690_MOESM2_ESM.pdf]

Additional file 2 – Complete CANDL specification of the case study

This document provides supplementary material for

- *D Gilbert, M Heiner, L Ghanbar, J Chodak: Spatial quorum sensing modelling using coloured hybrid Petri nets and simulative model checking; BMC Bioinformatics, Supplement issue: 12859-20-S4, DOI: 10.1186/s12859-019-2690-z*

The source file listed below can be downloaded from

- *http://www-dssz.informatik.tu-cottbus.de/DSSZ/Software/Examples*

The software tools required are available at

- *http://www-dssz.informatik.tu-cottbus.de/DSSZ/Software/Software*

In our toolkit, a coloured Petri net can either be specified in a graphical way using Snoopy or in a textual notation (written with any text editor), for which we use the Coloured Abstract Net Description Language (CANDL).

The following listing shows the complete CANDL specification of the coloured SPN developed in the manuscript and given there in Figure 6. The Petri net can be easily adjusted to a 1D, 2D or 3D scenario and conveniently configured for different conditions, such as dense or sparse bacterial colonies. By exchanging the keyword **colspn** with **colhpn**, the net will be read as a coloured HPN. Varying the boundary conditions or neighbourhood relation requires to adjust the colour functions.

For more details, please consult manuals and websites of our PetriNuts toolbox:

Manual coloured Petri nets F Liu, M Heiner and C Rohr: Manual for Colored Petri Nets in Snoopy; Technical report 02-12, Brandenburg University of Technology Cottbus, Department of Computer Science, March 2012.
http://www-dssz.informatik.tu-cottbus.de/publications/btu-reports/Manual_for_colored_Petri_nets_2012_03.pdf

Manual Marcie M Schwarick, C Rohr and M Heiner: Marcie Manual; Technical report 02-16, Brandenburg University of Technology Cottbus, Department of Computer Science, December 2016.
https://opus4.kobv.de/opus4-btu/frontdoor/index/index/docId/4056

Manual coloured hybrid Petri Nets M Herajy, F Liu, C Rohr and M Heiner: Coloured Hybrid Petri Nets in Snoopy - User Manual; Technical report 01-17, Brandenburg University of Technology Cottbus, Department of Computer Science, March 2017.
https://opus4.kobv.de/opus4-btu/frontdoor/index/index/docId/4157

```
colspn  [LiBiofilmV6]
{
constants:
all:
  int D = 1;
  int D1 = D;
  int D2 = D;
  int D3 = 1;
  int M1 = D1/2+1;
  int M2 = D2/2+1;
  int M3 = D3/2+1;
  int R = 0;
  int S1 = 1;
  int S2 = 1;
  int S3 = 1;
param: // kinetic constants
    double pfs_transl = 0.54;
    double pfs_transc = 2.1;
    double pfs_mRNA_d = 0.022;
    double b_reac_r = 0.01;
    double Spermi_uti = 0.0208;
    double SpeE_syn_r = 4.03e-06;
    double SRH_cleav = 0.00224;
    double SAM_Dec = 0.055;
    double SAH_Hydro_r = 0.00055;
    double Polyamine_uti = 0.15;
    double Pfs_prot_d = 0.003;
    double Methy_trans = 0.15;
    double Met_recov = 0.35;
    double MTR_syn_r = 2.21e-05;
    double MTR_e = 0.02;
    double LuxS_transl = 0.74;
    double LuxS_transc = 1.25;
    double LuxS_prot_d = 0.012;
    double LuxS_mRNA_d = 0.03;
    double DPD_deg_r = 0.005;
    double AdoMet_r = 0.962;
    double AI2_syn_r = 0.00067;
    double AI2_excret_r = 0.25;
    double k_open = 1;
    double k_close = 1;
    double k_transport = 0.001;
    double k_deg_LsrABCD = 0.0001;
    double k_phosphorylation = 0.001;
    double k_deg_LsrK = 0.0001;
    double k_biofilmformationAI = 0.001;
    double k_biofilmformationLsrR = 0.001;
    double k_transcription = 0.001;
    double k_deg_LsrR = 0.0001;
    double k_deg_LsrR_AI2_P = 0.0001;
    double k_basal_transcription = 0.0001;
    double k_incr_act = 0.001;
    double k_decr_act = 0.001;
    double k_diffusion = 0.001;

colorsets:
  Dot = {dot};
  CD1 = {1..D1};
  CD2 = {1..D2};
  CD3 = {1..D3};
  Distance = {0..D};
  Grid3D = PROD(CD1,CD2,CD3);

variables:
  CD1 : a; CD2 : b; CD3 : c;
  CD1 : x; CD2 : y; CD3 : z;
  CD1 :x0; CD2 :y0; CD3 : z0;

colorfunctions:
bool neighbour3D26(CD1 a,CD2 b,CD3 c,CD1 x,CD2 y,CD3 z) {
  (a=x-1 | a=x | a=x+1) & (b=y-1 | b=y | b=y+1)
   & (c=z-1 | c=z | c=z+1) & (!(a=x & b=y & c=z))
```

```
  & (1<=x & x<=D1) & (1<=y & y<=D2) & (1<=z & z<=D3) };

bool region(CD1 x,CD2 y,CD3 z,CD1 x0,CD2 y0,CD3 z0,
       Distance radius,Distance xd,Distance yd,Distance zd) {
   (x0-radius)<=x & x<=(x0+radius)
  & (y0-radius)<=y & y<=(y0+radius)
  & (z0-radius)<=z & z<=(z0+radius)
  & (1<=x & x<=D1) & (1<=y & y<=D2) & (1<=z & z<=D3)
  & (x%xd=0 & y%yd=0 & z%zd=0) };

places:
discrete:
  Grid3D go = [region(x,y,z,M1,M2,M3,R,S1,S2,S3)]0`(x,y,z);
  Grid3D AI2_In_Phase3
            = [region(x,y,z,M1,M2,M3,R,S1,S2,S3)]0`(x,y,z);
  Grid3D AI2_Out = [region(x,y,z,M1,M2,M3,R,S1,S2,S3)]0`(x,y,z);
  Grid3D AI2_P = [region(x,y,z,M1,M2,M3,R,S1,S2,S3)]0`(x,y,z);
  Grid3D Biofilm = [region(x,y,z,M1,M2,M3,R,S1,S2,S3)]0`(x,y,z);
  Grid3D LsrR_AI2_P
            = [region(x,y,z,M1,M2,M3,R,S1,S2,S3)]0`(x,y,z);
  Grid3D QSeBC = [region(x,y,z,M1,M2,M3,R,S1,S2,S3)]1`(x,y,z);
  Grid3D lsrGenes_LsrR
            = [region(x,y,z,M1,M2,M3,R,S1,S2,S3)]1`(x,y,z);
  Grid3D lsrGenes = [region(x,y,z,M1,M2,M3,R,S1,S2,S3)]0`(x,y,z);
  Grid3D LsrR = [region(x,y,z,M1,M2,M3,R,S1,S2,S3)]1`(x,y,z);
  Grid3D LsrK = [region(x,y,z,M1,M2,M3,R,S1,S2,S3)]1`(x,y,z);
  Grid3D LsrABCD = [region(x,y,z,M1,M2,M3,R,S1,S2,S3)]1`(x,y,z);
  Grid3D Biofilm_AI2
            = [region(x,y,z,M1,M2,M3,R,S1,S2,S3)]0`(x,y,z);
  Grid3D Biofilm_Lsr
            = [region(x,y,z,M1,M2,M3,R,S1,S2,S3)]0`(x,y,z);
  Grid3D pfs_gene = [region(x,y,z,M1,M2,M3,R,S1,S2,S3)]1`(x,y,z);
  Grid3D Spermidine
            = [region(x,y,z,M1,M2,M3,R,S1,S2,S3)]940`(x,y,z);
  Grid3D SRH = [region(x,y,z,M1,M2,M3,R,S1,S2,S3)]300`(x,y,z);
  Grid3D SAM = [region(x,y,z,M1,M2,M3,R,S1,S2,S3)]10000`(x,y,z);
  Grid3D SAH = [region(x,y,z,M1,M2,M3,R,S1,S2,S3)]320`(x,y,z);
  Grid3D Putrescine
            = [region(x,y,z,M1,M2,M3,R,S1,S2,S3)]4400`(x,y,z);
  Grid3D Pfs_prot
            = [region(x,y,z,M1,M2,M3,R,S1,S2,S3)]800`(x,y,z);
  Grid3D Pfs_mRNA
            = [region(x,y,z,M1,M2,M3,R,S1,S2,S3)]32`(x,y,z);
  Grid3D Nutrients
            = [region(x,y,z,M1,M2,M3,R,S1,S2,S3)]28350`(x,y,z);
  Grid3D Met = [region(x,y,z,M1,M2,M3,R,S1,S2,S3)]2248`(x,y,z);
  Grid3D MTR = [region(x,y,z,M1,M2,M3,R,S1,S2,S3)]180`(x,y,z);
  Grid3D MTA = [region(x,y,z,M1,M2,M3,R,S1,S2,S3)]296`(x,y,z);
  Grid3D LuxS_prot
            = [region(x,y,z,M1,M2,M3,R,S1,S2,S3)]400`(x,y,z);
  Grid3D LuxS_mRNA
            = [region(x,y,z,M1,M2,M3,R,S1,S2,S3)]16`(x,y,z);
  Grid3D LuxS_gene
            = [region(x,y,z,M1,M2,M3,R,S1,S2,S3)]1`(x,y,z);
  Grid3D Homocys
            = [region(x,y,z,M1,M2,M3,R,S1,S2,S3)]300`(x,y,z);
  Grid3D Decarb_SAM
            = [region(x,y,z,M1,M2,M3,R,S1,S2,S3)]220`(x,y,z);
  Grid3D DPD = [region(x,y,z,M1,M2,M3,R,S1,S2,S3)]300`(x,y,z);
  Grid3D Adenine
            = [region(x,y,z,M1,M2,M3,R,S1,S2,S3)]596`(x,y,z);
  Grid3D AI2_In_Phase2
            = [region(x,y,z,M1,M2,M3,R,S1,S2,S3)]60`(x,y,z);

transitions:
  close
 {[region(x,y,z,M1,M2,M3,R,S1,S2,S3)]}
    : [AI2_Out < {4000`(x,y,z)}]
    : [go - {(x,y,z)}]
    : MassAction(k_close)
    ;
  open
```

```
{[region(x,y,z,M1,M2,M3,R,S1,S2,S3)]}
   : [AI2_Out >= {4000`(x,y,z)}] & [go < {(x,y,z)}]
   : [go + {(x,y,z)}]
   : MassAction(k_open)
   ;
 BiofilmFormation_AI2
{[region(x,y,z,M1,M2,M3,R,S1,S2,S3)]}
   :
   : [Biofilm + {(x,y,z)}] & [QSeBC + {(x,y,z)}] & [AI2_In_Phase3 +
       {(x,y,z)}] & [Biofilm_AI2 + {(x,y,z)}] & [QSeBC - {(x,y,z)}] &
        [AI2_In_Phase3 - {(x,y,z)}]
   : MassAction(k_biofilmformationAI)
   ;
 BiofilmFormation_LsrR
{[region(x,y,z,M1,M2,M3,R,S1,S2,S3)]}
   :
   : [LsrR + {(x,y,z)}] & [QSeBC + {(x,y,z)}] & [Biofilm + {(x,y,z)}]
       & [AI2_In_Phase3 + {(x,y,z)}] & [Biofilm_Lsr + {(x,y,z)}] & [
       LsrR - {(x,y,z)}] & [QSeBC - {(x,y,z)}] & [AI2_In_Phase3 - {(x
       ,y,z)}]
   : MassAction(k_biofilmformationLsrR)
   ;
 Phosphorylation
{[region(x,y,z,M1,M2,M3,R,S1,S2,S3)]}
   :
   : [LsrK + {(x,y,z)}] & [AI2_P + {(x,y,z)}] & [AI2_In_Phase3 - {(x,
       y,z)}] & [LsrK - {(x,y,z)}]
   : MassAction(k_phosphorylation)
   ;
 TranscribeTranslate
{[region(x,y,z,M1,M2,M3,R,S1,S2,S3)]}
   :
   : [LsrK + {(x,y,z)}] & [LsrABCD + {(x,y,z)}] & [lsrGenes + {(x,y,z
       )}] & [LsrR + {(x,y,z)}] & [lsrGenes - {(x,y,z)}]
   : MassAction(k_transcription)
   ;
 Transport_in
{[region(x,y,z,M1,M2,M3,R,S1,S2,S3)]}
   : [go >= {(x,y,z)}]
   : [AI2_In_Phase3 + {(x,y,z)}] & [LsrABCD + {(x,y,z)}] & [AI2_Out -
        {(x,y,z)}] & [LsrABCD - {(x,y,z)}]
   : MassAction(k_transport)
   ;
 LsrABCD_deg
{[region(x,y,z,M1,M2,M3,R,S1,S2,S3)]}
   :
   : [LsrABCD - {(x,y,z)}]
   : MassAction(k_deg_LsrABCD)
   ;
 LsrK_deg
{[region(x,y,z,M1,M2,M3,R,S1,S2,S3)]}
   :
   : [LsrK - {(x,y,z)}]
   : MassAction(k_deg_LsrK)
   ;
 derepress
{[region(x,y,z,M1,M2,M3,R,S1,S2,S3)]}
   :
   : [lsrGenes + {(x,y,z)}] & [LsrR_AI2_P + {(x,y,z)}] & [
       lsrGenes_LsrR - {(x,y,z)}] & [AI2_P - {(x,y,z)}]
   : MassAction(k_incr_act)
   ;
 repress
{[region(x,y,z,M1,M2,M3,R,S1,S2,S3)]}
   :
   : [lsrGenes_LsrR + {(x,y,z)}] & [lsrGenes - {(x,y,z)}] & [LsrR -
       {(x,y,z)}]
   : MassAction(k_decr_act)
   ;
 LsrR_deg
{[region(x,y,z,M1,M2,M3,R,S1,S2,S3)]}
   :
```

```
    : [LsrR - {(x,y,z)}]
    : MassAction(k_deg_LsrR)
    ;
 BasalProduceLsrK
{[region(x,y,z,M1,M2,M3,R,S1,S2,S3)]}
    :
    : [LsrK + {(x,y,z)}]
    : MassAction(k_basal_transcription)
    ;
 BasalProduceLsrABCD
{[region(x,y,z,M1,M2,M3,R,S1,S2,S3)]}
    :
    : [LsrABCD + {(x,y,z)}]
    : MassAction(k_basal_transcription)
    ;
 LsrR_AI2_P_deg
{[region(x,y,z,M1,M2,M3,R,S1,S2,S3)]}
    :
    : [LsrR_AI2_P - {(x,y,z)}]
    : MassAction(k_deg_LsrR_AI2_P)
    ;
 BasalProduceLsrR
{[region(x,y,z,M1,M2,M3,R,S1,S2,S3)]}
    :
    : [LsrR + {(x,y,z)}]
    : MassAction(k_basal_transcription)
    ;
 pfs_translation
{[region(x,y,z,M1,M2,M3,R,S1,S2,S3)]}
    :
    : [Pfs_prot + {(x,y,z)}] & [Pfs_mRNA + {(x,y,z)}] & [Pfs_mRNA - {(
       x,y,z)}]
    : pfs_transl*Pfs_mRNA
    ;
 pfs_transcription
{[region(x,y,z,M1,M2,M3,R,S1,S2,S3)]}
    :
    : [Pfs_mRNA + {(x,y,z)}] & [pfs_gene + {(x,y,z)}] & [pfs_gene - {(
       x,y,z)}]
    : pfs_transc
    ;
 bio_reac
{[region(x,y,z,M1,M2,M3,R,S1,S2,S3)]}
    :
    : [Met + {(x,y,z)}] & [Nutrients - {(x,y,z)}]
    : b_reac_r*Nutrients
    ;
 Spermi_util
{[region(x,y,z,M1,M2,M3,R,S1,S2,S3)]}
    :
    : [Spermidine - {(x,y,z)}]
    : Spermi_uti*Spermidine
    ;
 SpeE_syn
{[region(x,y,z,M1,M2,M3,R,S1,S2,S3)]}
    :
    : [MTA + {(x,y,z)}] & [Spermidine + {(x,y,z)}] & [Decarb_SAM - {(x
       ,y,z)}] & [Putrescine - {(x,y,z)}]
    : SpeE_syn_r*Decarb_SAM*Putrescine
    ;
 SRH_cleave
{[region(x,y,z,M1,M2,M3,R,S1,S2,S3)]}
    :
    : [LuxS_prot + {(x,y,z)}] & [Homocys + {(x,y,z)}] & [DPD + {(x,y,z
       )}] & [LuxS_prot - {(x,y,z)}] & [SRH - {(x,y,z)}]
    : SRH_cleav*LuxS_prot*SRH
    ;
 SAM_Decarb
{[region(x,y,z,M1,M2,M3,R,S1,S2,S3)]}
    :
    : [Decarb_SAM + {(x,y,z)}] & [SAM - {(x,y,z)}]
    : SAM_Dec*SAM
```

```
    ;
 SAH_Hydro
{[region(x,y,z,M1,M2,M3,R,S1,S2,S3)]}
    :
    : [SRH + {(x,y,z)}] & [Adenine + {(x,y,z)}] & [Pfs_prot + {(x,y,z)
        }] & [SAH - {(x,y,z)}] & [Pfs_prot - {(x,y,z)}]
    : SAH_Hydro_r*SAH*Pfs_prot
    ;
 Polyamine_util
{[region(x,y,z,M1,M2,M3,R,S1,S2,S3)]}
    :
    : [Adenine - {(x,y,z)}]
    : Polyamine_uti*Adenine
    ;
 Pfs_prot_deg
{[region(x,y,z,M1,M2,M3,R,S1,S2,S3)]}
    :
    : [Pfs_prot - {(x,y,z)}]
    : Pfs_prot_d*Pfs_prot
    ;
 Pfs_mRNA_deg
{[region(x,y,z,M1,M2,M3,R,S1,S2,S3)]}
    :
    : [Pfs_mRNA - {(x,y,z)}]
    : pfs_mRNA_d*Pfs_mRNA
    ;
 MethyTrans
{[region(x,y,z,M1,M2,M3,R,S1,S2,S3)]}
    :
    : [SAH + {(x,y,z)}] & [SAM - {(x,y,z)}]
    : Methy_trans*SAM
    ;
 Met_recover
{[region(x,y,z,M1,M2,M3,R,S1,S2,S3)]}
    :
    : [Met + {(x,y,z)}] & [Homocys - {(x,y,z)}]
    : Met_recov*Homocys
    ;
 MTR_syn
{[region(x,y,z,M1,M2,M3,R,S1,S2,S3)]}
    :
    : [MTR + {(x,y,z)}] & [Adenine + {(x,y,z)}] & [Pfs_prot + {(x,y,z)
        }] & [MTA - {(x,y,z)}] & [Pfs_prot - {(x,y,z)}]
    : MTR_syn_r*Pfs_prot*MTA
    ;
 MTR_excret
{[region(x,y,z,M1,M2,M3,R,S1,S2,S3)]}
    :
    : [MTR - {(x,y,z)}]
    : MTR_e*MTR
    ;
 LuxS_translation
{[region(x,y,z,M1,M2,M3,R,S1,S2,S3)]}
    :
    : [LuxS_prot + {(x,y,z)}] & [LuxS_mRNA + {(x,y,z)}] & [LuxS_mRNA -
         {(x,y,z)}]
    : LuxS_transl*LuxS_mRNA
    ;
 LuxS_transcription
{[region(x,y,z,M1,M2,M3,R,S1,S2,S3)]}
    :
    : [LuxS_gene + {(x,y,z)}] & [LuxS_mRNA + {(x,y,z)}] & [LuxS_gene -
         {(x,y,z)}]
    : LuxS_transc
    ;
 LuxS_prot_deg
{[region(x,y,z,M1,M2,M3,R,S1,S2,S3)]}
    :
    : [LuxS_prot - {(x,y,z)}]
    : LuxS_prot_d*LuxS_prot
    ;
 LuxS_mRNA_deg
```

```
  {[region(x,y,z,M1,M2,M3,R,S1,S2,S3)]}
     :
     : [LuxS_mRNA - {(x,y,z)}]
     : LuxS_mRNA_d*LuxS_mRNA
     ;
  DPD_deg
  {[region(x,y,z,M1,M2,M3,R,S1,S2,S3)]}
     :
     : [DPD - {(x,y,z)}]
     : DPD_deg_r*DPD
     ;
  AdoMet
  {[region(x,y,z,M1,M2,M3,R,S1,S2,S3)]}
     :
     : [SAM + {(x,y,z)}] & [Met - {(x,y,z)}]
     : AdoMet_r*Met
     ;
  AI2_syn
  {[region(x,y,z,M1,M2,M3,R,S1,S2,S3)]}
     :
     : [AI2_In_Phase2 + {(x,y,z)}] & [DPD - {(x,y,z)}]
     : AI2_syn_r*DPD
     ;
  AI2_excret
  {[region(x,y,z,M1,M2,M3,R,S1,S2,S3)]}
     :
     : [AI2_Out + {(x,y,z)}] & [AI2_In_Phase2 - {(x,y,z)}]
     : AI2_excret_r*AI2_In_Phase2
     ;
  Diffusion
  {[neighbour3D26(x,y,z,a,b,c)]}
     :
     : [AI2_Out + {(a,b,c)}] & [AI2_Out - {(x,y,z)}]
     : MassAction(k_diffusion)
     ;

} // end colspn  [LiBiofilmV6]
```
