# Supplementary material for: Spatial quorum sensing modelling using coloured hybrid Petri nets and simulative model checking
Source: BMC Bioinformatics. 2019 Apr 18;20(Suppl 4):173. doi: 10.1186/s12859-019-2690-z (PMC6471779; doi:10.1186/s12859-019-2690-z)
Supplement: Supplementary file 3 — Supplementary material for model validation. Additional explanations and figures illustrating various aspects of the model validation performed. (PDF 3614 KB) [file 12859_2019_2690_MOESM3_ESM.pdf]

Additional file 3 – Supplementary material for model validation

This document provides supplementary material for

- *D Gilbert, M Heiner, L Ghanbar, J Chodak: Spatial quorum sensing modelling using coloured hybrid Petri nets and simulative model checking; BMC Bioinformatics, Supplement issue: 12859-20-S4, DOI: 10.1186/s12859-019-2690-z*

The source files used in this document can be downloaded from

- *http://www-dssz.informatik.tu-cottbus.de/DSSZ/Software/Examples*

The software tools required are available at

- *http://www-dssz.informatik.tu-cottbus.de/DSSZ/Software/Software*

For more details, please consult manuals and websites of our PetriNuts toolbox:

Manual coloured Petri nets F Liu, M Heiner and C Rohr: Manual for Colored Petri Nets in Snoopy; Technical report 02-12, Brandenburg University of Technology Cottbus, Department of Computer Science, March 2012. http://www-dssz.informatik.tu-cottbus.de/publications/btu-reports/Manual_for_colored_Petri_nets_2012_03.pdf

Manual Marcie M Schwarick, C Rohr and M Heiner: Marcie Manual; Technical report 02-16, Brandenburg University of Technology Cottbus, Department of Computer Science, December 2016. https://opus4.kobv.de/opus4-btu/frontdoor/index/index/docId/4056

Manual coloured hybrid Petri Nets M Herajy, F Liu, C Rohr and M Heiner: Coloured Hybrid Petri Nets in Snoopy - User Manual; Technical report 01-17, Brandenburg University of Technology Cottbus, Department of Computer Science, March 2017. https://opus4.kobv.de/opus4-btu/frontdoor/index/index/docId/4157

To obtain more information re the Petri net analyser Charlie, explore:

- M Heiner, M Schwarick and J Wegener: Charlie – an extensible Petri net analysis tool; In Proc. PETRI NETS 2015, Brussels, Springer, LNCS, volume 9115, pages 200?211, June 2015. http://link.springer.com/chapter/10.1007/978-3-319-19488-2_10
- MA Blätke, M Heiner, and W Marwan: Tutorial - Petri Nets in Systems Biology; Technical report, Otto von Guericke University Magdeburg, Magdeburg Centre for Systems Biology, August 2011. http://www-dssz.informatik.tu-cottbus.de/publications/tutorial_heidelberg_2011/Tutorial_English.pdf

Phase2 model validation

We consider the model with an appropriate environment ensuring infinite in/outflow. This is achieved by adding two source transitions and one sink transition, making the Petri net transition-bordered; i.e., there are no sink/source places anymore; compare Figure 1. Structural analysis was performed with Charlie, see the abridged log file below for the main results, and Figure 2 for a visualisation of the T-invariants, which was done with Snoopy.

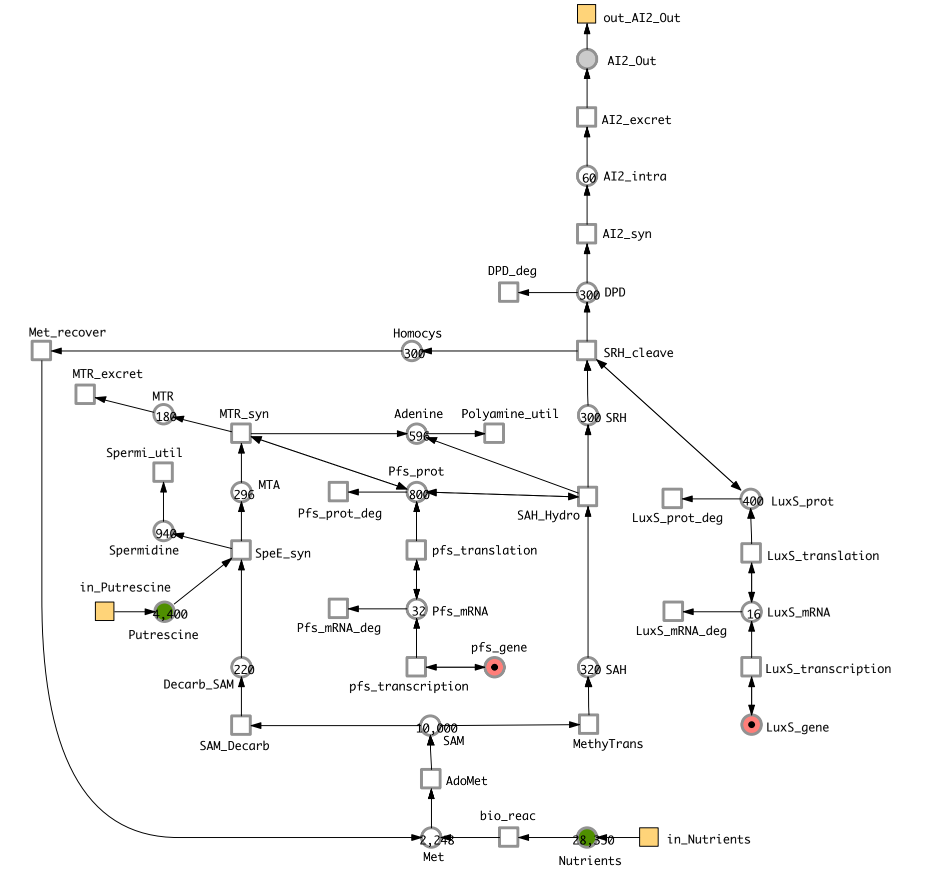

Figure 1 Phase2 model with environment. The three transitions added to model infinite in/outflow are given in yellow. See Figure 2 for the T-invariants.

```
Charlie (v2.0-b212-r8932) - Li2006-env.andl

number of places: 21
number of transitions: 26
number of arcs: 56

input places:
    no input places
output places:
    no output places
input transitions:
        |24.in_Nutrients        :1,
        |25.in_Putrescine       :1
output transitions:
        |3.DPD_deg      :1,
        |4.LuxS_mRNA_deg        :1,
```

```
        |5.LuxS_prot_deg          :1,
        |8.MTR_excret    :1,
        |12.Pfs_mRNA_deg          :1,
        |13.Pfs_prot_deg          :1,
        |14.Polyamine_util        :1,
        |19.Spermi_util :1,
        |23.out_AI2_Out :1
```

Applying rule:

```
!FT0 => !k-B & !SB & !1-B
!k-B => !SB
!k-B => !1-B
!SB => !CPI
```

Analyzer: InvariantAnalyzer

```
net is NOT covered by P-Invariants (CPI) because of:
        |0.AI2_intra     :1,
        |1.AI2_Out       :1,
        |2.Adenine       :1,
        |3.DPD  :1,
        |4.Decarb_SAM    :1,
        |5.Homocys       :1,
        |7.LuxS_mRNA     :1,
        |8.LuxS_prot     :1,
        |9.MTA  :1,
        |10.MTR :1,
        |11.Met :1,
        |12.Nutrients    :1,
        |13.Pfs_mRNA     :1,
        |14.Pfs_prot     :1,
        |15.Putrescine   :1,
        |16.SAH :1,
        |17.SAM :1,
        |18.SRH :1,
        |19.Spermidine   :1
minimal semipositive place invariants: 2

net is covered by T-Invariants
minimal semipositive transition invariants: 7
```

Analyzer: SiphonAnalyzer

```
STP is valid
    siphon:
        |20.pfs_gene     :1
        maximal trap:
        |20.pfs_gene     :1
        is sufficiently marked
    siphon:
        |6.LuxS_gene     :1
        maximal trap:
        |6.LuxS_gene     :1
        is sufficiently marked
2 siphons computed
```

Applying rule:

```
STP & HOM & NMB & ES => LIV
LIV => ! DSt & ! DTr
```

FINAL RESULTS

PUR	ORD	HOM	NBM	CSV	SCF	FT0	TF0	FP0	PF0	CON	SC	NC
N	Y	Y	Y	N	N	N	N	Y	Y	Y	N	ES

RKTH	STP	CPI	CTI	SCTI	SB	k-B	1-B	DCF	DSt	DTr	LIV	REV
–	Y	N	Y	–	N	N	N	–	0	Y	Y	–

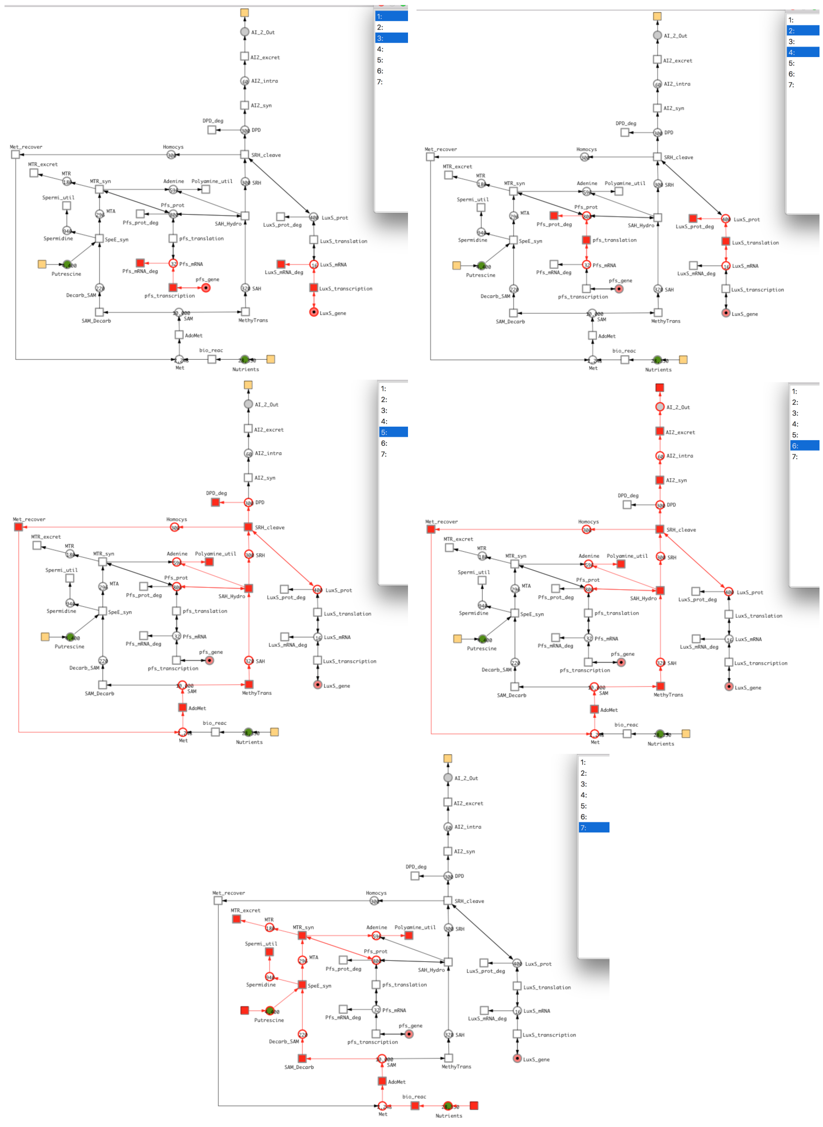

Figure 2 Phase2 model with environment – T-invariants. The net is covered with T-invariants. T-invariants were computed with Charlie, and visualised with Snoopy; compare Figure 1.

Having gained some confidence in the model by structural analysis we continued with simulative model checking over the deterministic traces of all species; see Figure 3 for the determined categories.

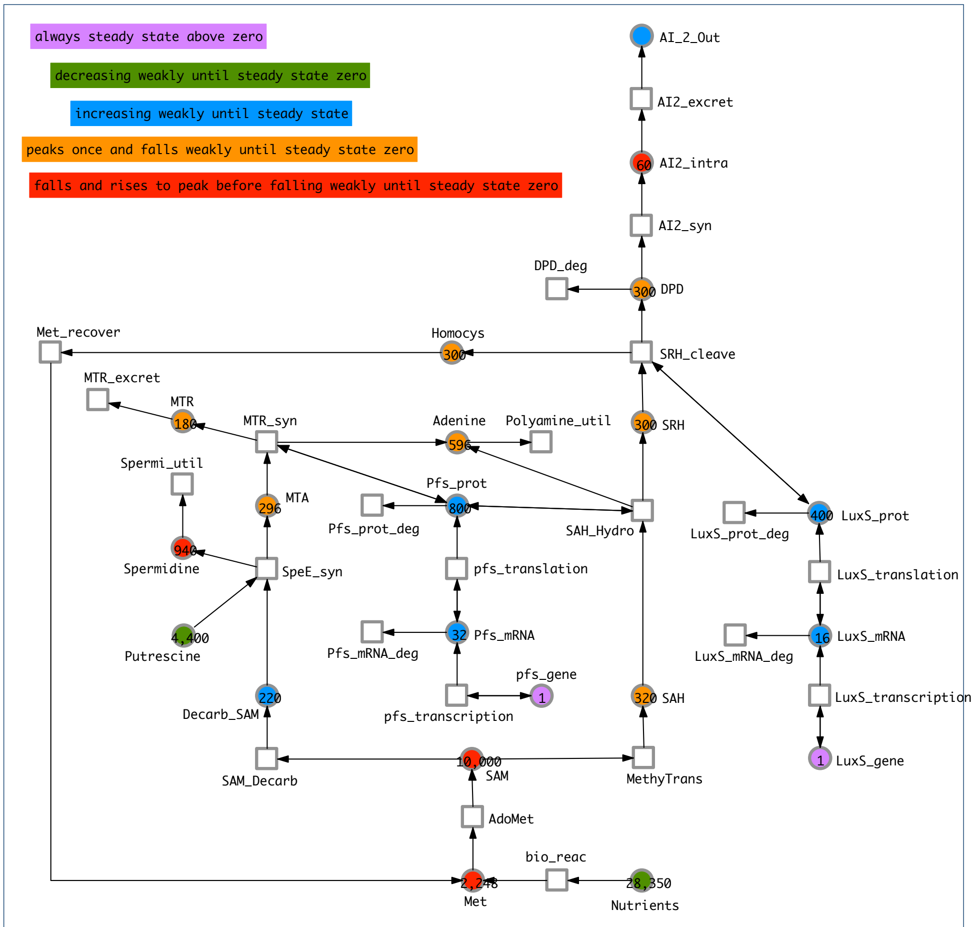

Figure 3 Validating Phase 2 model. Categories as determined by simulative model checking are highlighted in different colours.

Phase3 model validation

We consider the model with an appropriate environment ensuring infinite in/outflow. This is achieved by adding one sink transition, making the Petri net transition-bordered; i.e., there are no sink/source places anymore; compare Figure 4. Structural analysis was performed with Charlie, see the abridged log file below for the main results, and Figure 5 for a visualisation of the T-invariants, which was done with Snoopy.

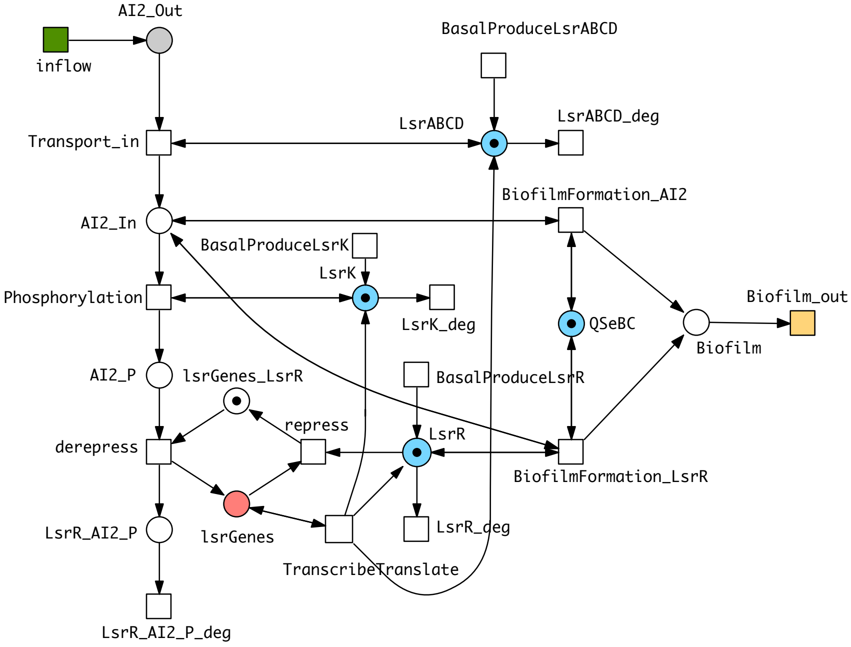

Figure 4 Phase3 model with environment, but without go mechanism. One sink transition was added to model infinite in/outflow, given in yellow. See Figure 5 for the T-invariants.

```
Charlie (v2.0-b212-r8932) - Phase3_BiofilmV5-env-noGo.andl

number of places: 11
number of transitions: 16
number of arcs: 41

input places:
    no input places
output places:
    no output places
input transitions:
        |10.BasalProduceLsrK     :1,
        |11.BasalProduceLsrABCD  :1,
        |13.BasalProduceLsrR     :1,
        |14.inflow       :1
output transitions:
        |5.LsrABCD_deg   :1,
        |6.LsrK_deg      :1,
        |9.LsrR_deg      :1,
        |12.LsrR_AI2_P_deg       :1,
        |15.out2         :1

Applying rule:
!FT0 => !k-B & !SB & !1-B
!k-B => !SB
!k-B => !1-B
!SB => !CPI

Analyzer: InvariantAnalyzer

net is NOT covered by P-Invariants (CPI) because of:
        |0.AI2_Out       :1,
        |1.LsrABCD       :1,
        |2.AI2_In        :1,
        |3.LsrK :1,
        |5.AI2_P         :1,
        |7.LsrR :1,
        |9.LsrR_AI2_P    :1,
        |10.Biofilm      :1
minimal semipositive place invariants: 2

net is covered by T-Invariants
minimal semipositive transition invariants: 8

Analyzer: SiphonAnalyzer

STP is valid
    siphon:
        |4.QSeBC         :1
        maximal trap:
        |4.QSeBC         :1
        is sufficiently marked
    siphon:
        |6.lsrGenes_LsrR         :1,
        |8.lsrGenes      :1
        maximal trap:
        |6.lsrGenes_LsrR         :1,
        |8.lsrGenes      :1
        is sufficiently marked
2 siphons computed
```

FINAL RESULTS

PUR	ORD	HOM	NBM	CSV	SCF	FT0	TF0	FP0	PF0	CON	SC	NC
N	Y	Y	Y	N	N	N	N	Y	Y	Y	N	nES

RKTH	STP	CPI	CTI	SCTI	SB	k–B	1–B	DCF	DSt	DTr	LIV	REV
–	Y	N	Y	–	N	N	N	–	0	–	–	–

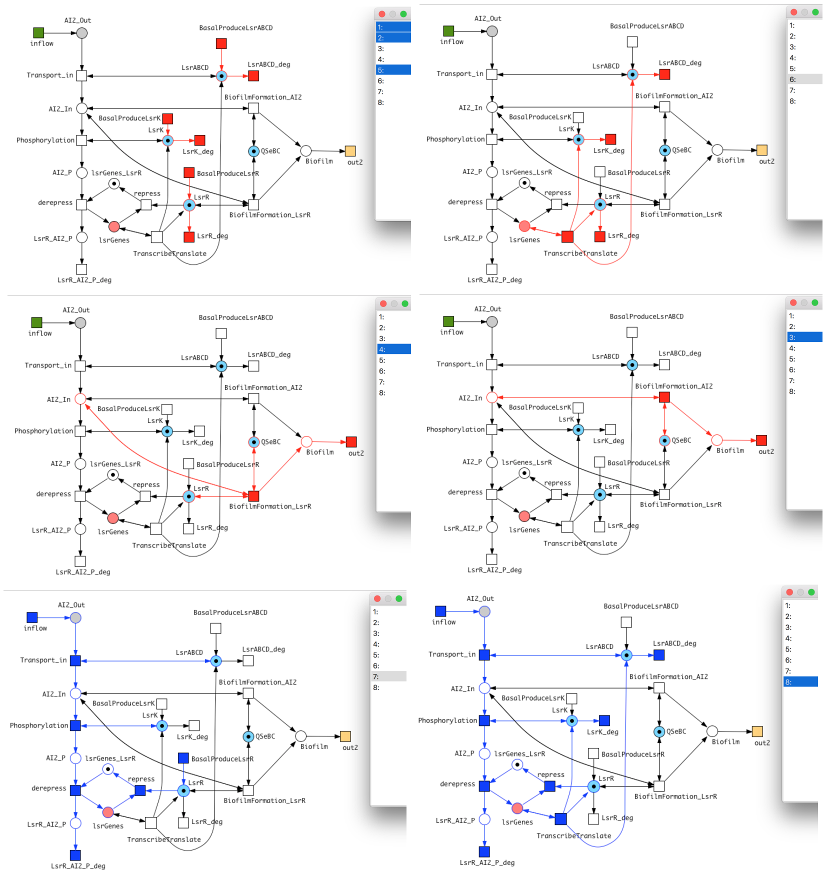

Figure 5 Phase3 model with environment – T-invariants. The net is covered with T-invariants. T-invariants were computed with Charlie, and visualised with Snoopy; compare Figure 4.

Validation of the non-spatial combined model

The structural analysis of the non-spatial combined model with appropriate in/out-flow, see Figure 6, is straightforward and free of surprises. The model is a composition of the two net components given in Figure 1 and Figure 4 by merging the two places *AI2_Out*, complemented by an abstract diffusion transition. The combined model inherits - due to the simple interface between the two components – the weaker properties of the components' properties, and the P- und T-invariants are a combination of the P- and T-invariants of the components. Figure 7 shows the two T-invariants, bridging both components, which are combinations of the T-invariant 6 in Figure 2 with the T-invariants 7, 8 in Figure 5.

——————————FINAL RESULTS——————————

PUR	ORD	HOM	NBM	CSV	SCF	FT0	TF0	FP0	PF0	CON	SC	NC
N	Y	Y	Y	N	N	N	N	Y	Y	Y	N	nES
RKTH	STP	CPI	CTI	SCTI	SB	k–B	1–B	DCF	DSt	DTr	LIV	REV
–	Y	N	Y	–	N	N	N	–	0	–	–	–

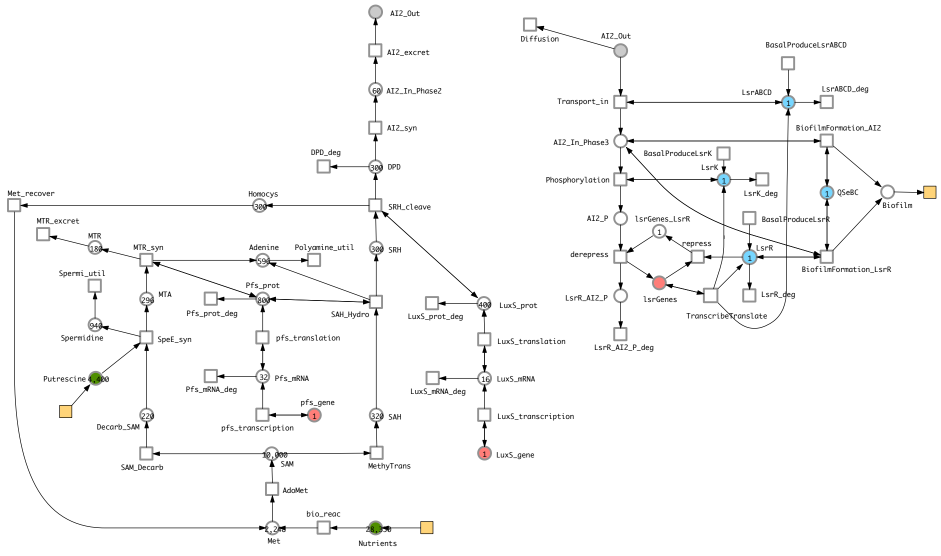

Figure 6 Non-spatial combined model with environment, but without go mechanism. Any transitions added to obtain infinite in/outflow are given in yellow. This model is a composition of the two net components given in Figure 1 and Figure 4 by merging the two places *AI2_Out*. See Figure 7 for its T-invariants.

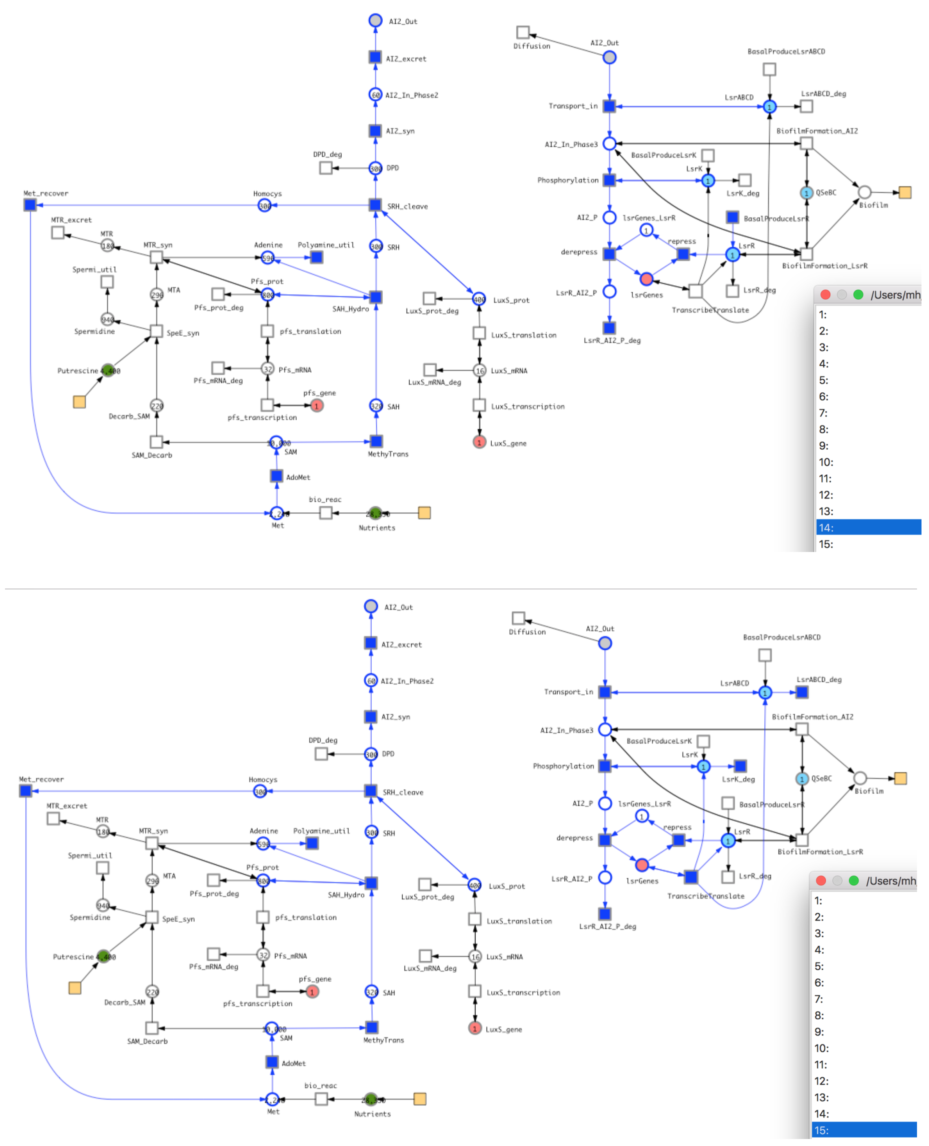

Figure 7 Non-spatial combined model with environment – T-invariants. The net is covered with T-invariants, which are – due to the simple interface between the two components – just a combination of the T-invariants of both components; compare Figures 2, 5. Shown are the two T-invariants, bridging both components. T-invariants were computed with Charlie, and visualised with Snoopy; compare Figure 6.
